# The effects of berberine supplementation on cardiovascular risk factors in adults: A systematic review and dose-response meta-analysis

**DOI:** 10.3389/fnut.2022.1013055

**Published:** 2022-10-14

**Authors:** Mohammad Zamani, Mahtab Zarei, Mahlagha Nikbaf-Shandiz, Shabnam Hosseini, Farideh Shiraseb, Omid Asbaghi

**Affiliations:** ^1^Department of Clinical Nutrition, School of Nutritional Sciences and Dietetics, Tehran University of Medical Sciences, Tehran, Iran; ^2^Department of Cellular and Molecular Nutrition, School of Nutritional Sciences and Dietetics, Tehran University of Medical Sciences (TUMS), Tehran, Iran; ^3^Student Research Committee, Tabriz University of Medical Sciences, Tabriz, Iran; ^4^Nutrition and Endocrine Research Center, Research Institute for Endocrine Sciences, Shahid Beheshti University of Medical Sciences, Tehran, Iran; ^5^Department of Community Nutrition, School of Nutritional Sciences and Dietetics, Tehran University of Medical Sciences (TUMS), Tehran, Iran; ^6^Cancer Research Center, Shahid Beheshti University of Medical Sciences, Tehran, Iran; ^7^Student Research Committee, Shahid Beheshti University of Medical Sciences, Tehran, Iran

**Keywords:** berberine, cardiovascular risk factors, systematic review, meta-analysis, adult

## Abstract

Cardiovascular disease (CVD) is a major concern today. Herbal medicine is one helping way to control CVD risks. One conclusive of herbal medicine is Berberine (BBR) and converse about it still exists, to clarify this issue, this meta-analysis was performed. PubMed/Medline, Scopus, and Web of Science were searched for RCTs in adults on the effect of BBR supplementation on CVD risk factors up to July 2022. The pooled results showed BBR significantly reduced triglyceride (WMD = −23.70 mg/dl; 95%CI −30.16, −17.25; *P* < 0.001), total cholesterol (WMD = −20.64 mg/dl; 95%CI −23.65, −17.63; *P* < 0.001), low-density lipoprotein WMD = −9.63 mg/dl; 95%CI, −13.87, −5.39; *P* < 0.001), fasting blood glucose (FBG) (WMD = −7.74 mg/dl; 95%CI −10.79, −4.70; *P* < 0.001), insulin (WMD = −3.27 mg/dl; 95%CI −4.46,−2.07; *P* < 0.001), HbA1c (WMD = −0.45%; 95%CI −0.68, −0.23; *P* < 0.001), HOMA-IR (WMD = −1.04; 95%CI −1.55, −0.52; *P* < 0.001), systolic blood pressure (WMD = −5.46 mmHg; 95%CI −8.17, −2.76; *P* < 0.001), weight (WMD = −0.84; 95%CI −1.34,−0.34; *P* < 0.001), body mass index (WMD = −0.25 kg/m^2^; 95%CI −0.46, −0.04; *P* = 0.020), while increased high-density lipoprotein (HDL) (WMD = 1.37 mg/dl; 95%CI 0.41,2.23; *P* = 0.005). The optimal dose of BBR was 1 g/day for TG, TC, and weight, 1.8 g/day for insulin and HOMA-IR, and 5 g/day for HDL. FBG's most efficient time frame was 40 weeks from the beginning of supplementation, whereas DBP and waist circumference was 50 weeks. In conclusion, the lipid profile, FBG balance, obesity parameters, and SBP were improved with BBR supplementation.

**Systematic review registration:**

CRD42022347004.

## Introduction

Cardiovascular disease (CVD), a general term for heart and blood vessel disorders, is still the first-ranked cause of death despite recent advances in its management (1). World Health Organization (WHO) has noted that ~19.7 million people die every year due to CVDs, which equals 32 % of all deaths globally (1). Common cardiovascular risk factors, such as dyslipidemia and hyperglycemia, are mainly influenced by modifiable lifestyle and dietary factors (2, 3). To date, the cardio-protective effects of many dietary patterns, food groups, and functional foods have been investigated (4).

Traditional herbs have gained more attention since they are often cheaper, more locally available, with fewer side effects than synthetic drugs. Berberine (BBR), a plant alkaloid with known pharmacological properties extracted from Chinese traditional herbs (5), has been the subject of more research about its ameliorative effect on CVD risk factors (6); through suggested mechanisms (7). The major risk factors for CVD are well-established and they include metabolic syndrome components (dyslipidemia, hypertension, diabetes or insulin resistance, and abdominal obesity), inflammatory markers, and liver enzymes (8). These risk factors contribute to future CVD, stroke, diabetes, and mortality in individuals (8). BBR supplementation could be effective in either primary prevention or secondary prevention of CVD (6, 9, 10). Preclinical (animal, *in vitro*) studies demonstrate that BBR has positive effects on lowering blood lipids, blood glucose, and controlling weight and blood pressure (10, 11). Previous meta-analyses have been conducted on the effect of BBR administration on CVD risk factors, but they are not comprehensive and conclusive. BBR supplementation ameliorated MetS components (dyslipidemia, insulin resistance, hypertension, obesity) in previous meta-analyses (12–16). In addition, the effect of BBR supplementation on other CVD risk factors such as inflammatory markers and liver enzymes has been assessed by other meta-analyses (13, 17, 18). These meta-analyses have either assessed a single MetS component (12, 17), showed null/inconclusive results at the end (13, 14, 19), or included a few studies in their meta-analysis (12, 14).

Therefore, because the existing literature still lacks an appropriate comprehensive answer to whether BBR is effective on CVD risk factors or not, with finding the optimal dose and duration, we aimed to perform a novel comprehensive dose-response meta-analysis on the effect of BBR on all CVD risk factors in adults.

## Materials and methods

### Search strategy and study selection

The current study was reported according to the Preferred Reporting Items for Systematic Reviews and Meta-Analysis (PRISMA) (20). The protocol has been registered at PROSPERO (CRD42022347004).

**Figure 1:**
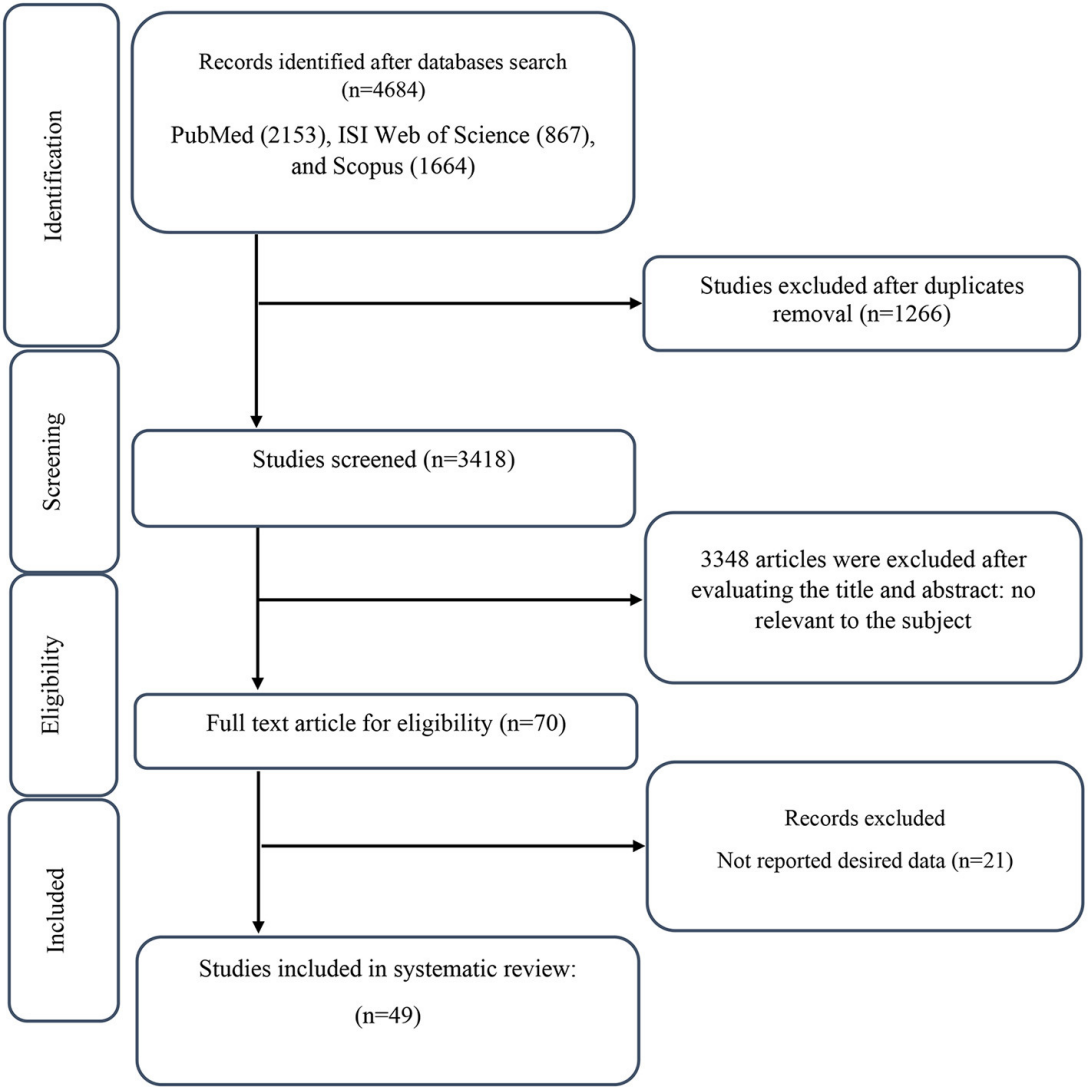
Flow chart of study selection for inclusion trials in the systematic review.

We conducted a systematic literature search in the following databases without any time, length of study, or language restrictions: PubMed/Medline, Scopus, Web of Science, EMBASE, the Cochrane databases, and Google Scholar (all of them up to July 2022). The framework that we used for our search was the PICO (Participant, Intervention, Comparison/Control, Outcome) strategy, which is recommended by Cochrane: (1) participants; (2) intervention group (which was treated by BBR); (3) comparison/Control group (non-BBR supplementation), and (4) outcome (all of the CVD risk factors that will be mentioned in inclusion criteria section). The full search strategy and the terms used to search in each database could be found in detail in Figure 1. We additionally screened the reference lists of previous systematic reviews and meta-analyses in order not to miss any related studies. To make sure that no studies were overlooked, we started the data collection process by using a combination of MeSH terms and keywords. The following keywords were manually used to search all related study reference lists: berberine OR huangliansu OR berberinum OR Xiaopojian OR barberry OR “Berberis vulgaris” OR Berberis) AND (Intervention OR “Intervention Study” OR “Intervention Studies” OR Randomized OR Random OR Randomly OR Placebo OR “Clinical Trial” OR Trial OR Trials OR “Randomized Clinical Trial” OR RCT OR blinded OR “double blind” OR “double blinded” “Controlled Trial” “Randomized Controlled Trial” OR “Controlled Clinical Trial” OR “Pragmatic Clinical Trial” OR “Cross-Over Studies” OR “Cross-Over” OR “Cross-Over Study” OR Parallel OR “Parallel Study” OR “Parallel trial”).

### Study selection

To include clinical studies, we considered the following criteria: (1) Only randomized clinical trials (parallel or crossover). (2) The intervention duration ≥1 week; (3) Studies with adult human subjects (≥18 years); (5) Studies that reported mean ± standard deviation (SD) or other effect sizes from which the calculation of mean and SD was possible; (6) Studies that examined the effect of BBR supplementation on triglyceride (TG), total cholesterol (TC), low-density lipoprotein (LDL), and high-density lipoprotein (HDL), fasting blood glucose, hemoglobin A1c (HbA1c), serum insulin, homeostasis model assessment-insulin resistance (HOMA-IR), systolic blood pressure (SBP), diastolic blood pressure (DBP), C-reactive protein (CRP), interleukin-6, (IL-6), weight, waist circumference (WC), body mass index (BMI), aspartate transaminase (AST) and alanine transaminase (ALT). The exclusion criteria were as follows: (1) studies on animal subjects and *in-vitro* studies; (2) studies on children and adolescents; (3) grey literature, reviews, conference abstracts, editorials, and books; (4) RCTs that did not have control/placebo groups.

### Data collection

MZ and OA independently screened the titles and abstracts of studies and discussed them with each other in case of any disagreement. Then, after re-assessment, MZ and OA extracted the following information from the included studies: first author's name, year of publication, country, type of clinical trial, participant characteristics (mean age, BMI, sex), duration of intervention, randomization, blinding, adverse effects sample size, the number of participants in the intervention and control groups, form and dosage of supplemented BBR, the health status of participants (healthy, diabetes, etc.), and outcome values. To solve any inconsistency in dosage, all of the BBR supplementation doses were converted to g/day.

### Assessment of studies quality

Included studies were screened for any source of bias, including random sequence generation, allocation concealment, participant and staff blindness, outcome assessor blinding, incomplete outcome data, selective reporting, and other biases using the Cochrane Collaboration tool (21) (**Table 2**). Then, three groups of high (general high risk > 2 high risk), moderate (general moderate risk = 2 high risk), and low (general low risk < 2 high risk) risk of bias were defined. Two reviewers (MZ and OA) independently assessed the quality of the work, and any conflicting opinions were settled by discussion.

### Assessment of certainty

The GRADE (Grading of Recommendations Assessment, Development, and Evaluation) approach was used to assess and summarize the certainty of evidence among the included studies, as described previously (**Table 4**) (22).

### Statistical analysis

Statistical analyses were conducted using Stata version 11.0 (Stata Corp, College Station, TX). All tests were two-tailed with *P*-values < 0.05 considered statistically significant. The pooled weighted mean difference (WMD) was calculated using a random-effects model (23). Mean differences in our outcomes were calculated from baseline to the after-intervention period between BBR supplementation and control groups. The SD of the mean difference was calculated using the following formula: SD = square root [(SD at baseline)^2^+ (SD at the end of study)^2^ –(2 r × SD at baseline × SD at the end of study)] (24). In each study reporting standard errors (SEs), 95 percent confidence intervals (CIs), or interquartile ranges (IQRs), to transform these values into SDs, we used Hozo et al. approach. We used the [SD = SE × √*n* (*n* = the number of individuals in each group)] formula to calculate SD (25). A correlation coefficient of 0.8 was used for r (26). After visual inspection of forest plots or Cochrane's Q test (27), heterogeneity was evaluated using the I square (I^2^) statistic (*P* = 0.05 and I^2^ > 40%) (28). Subgroup analysis was performed to explore the source of heterogeneity. Subgroups were selected based on the required minimum number of studies according to the criteria set by Fu et al., where there should be at least 6–10 studies for continuous and a minimum of 4 studies for categorical subgroup variables (29, 30). Subgroup analyses were performed regarding BBR dosage (≤1 g/d and >1 g/d), intervention duration (≤ 8 weeks and >8 weeks) sex (male, female), health status [dyslipidemia, type 2 diabetes, Metabolic Syndrome, non-alcoholic fatty liver disease (NAFLD)], baseline of TG (<150, ≥150 mg/dl), TC (<200, ≥200 mg/dl), LDL (<100, ≥100 mg/dl), HDL (<40, ≥40 mg/dl), FBG (<100, ≥100 mg/dl), SBP (<120, ≥120 mmHg), DBP (<80, ≥80 mmHg), and baseline BMI [normal (18.5–24.9 kg/m^2^), overweight (25–29.9 kg/m^2^) and obese (>30 kg/m^2^)], and category of risk of bias in studies (high, moderate, low). Studies examining the impact of BBR supplementation on CVD risk variables used the Begg's and Eager tests according to the number of studies for every outcome and the funnel plot test to evaluate publication bias (31, 32). Using the leave-one-out technique (removing one trail at a time and recalculating the impact size), we have done sensitivity analysis to establish how many inferences were dependent on a single sample to examine each study's impact on the pooled effect size (33). The possible impact of BBR (g/d) dose and duration on CVD risk variables was evaluated using meta-regression. In order to evaluate the effect of BBR supplementation on CVD risk variables, we also employed a non-linear model to include the associated dose-response data from several trials (34, 35).

## Results

### Study selection

The selection process of the included studies is presented in Figure 1. A database search resulted in identifying a total of 4,684 studies, including PubMed (*n* = 2,153), ISI Web of Science (*n* = 867), and Scopus (*n* = 1,664). A total of 1,266 duplicated studies were excluded, and 3,418 studies were screened based on title and abstract. After screening, 3,348 irrelevant studies were excluded, and 70 full-text studies were considered. In the end, 21 studies were excluded due to reporting non-desired outcomes. As a result, 49 studies were included in the systematic and meta-analysis review (36–84).

### Study characteristics

**Figure 2:**
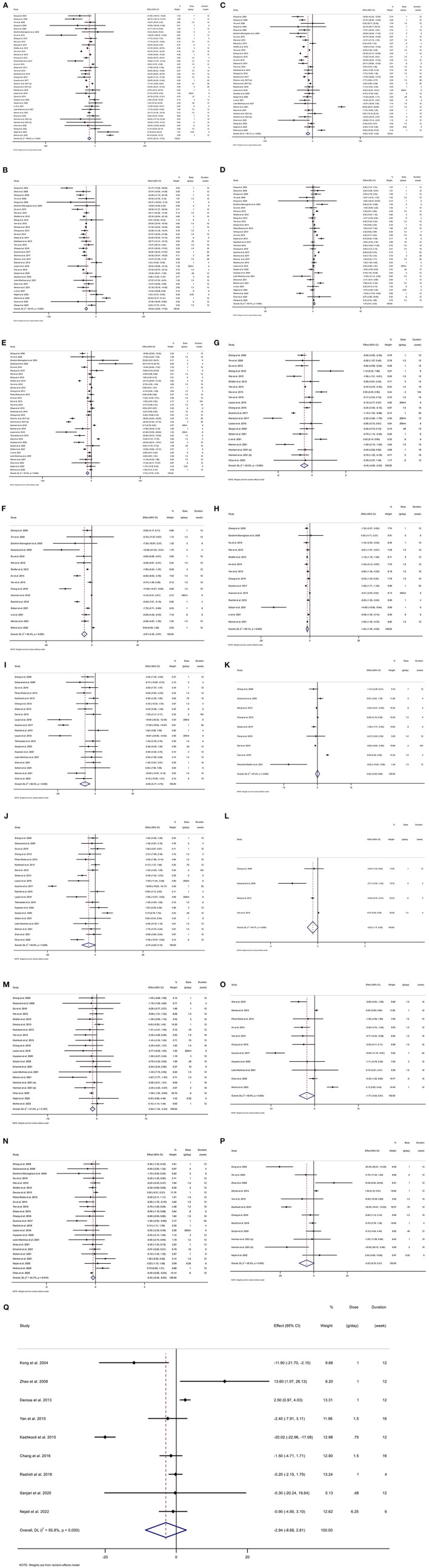
Forest plot detailing weighted mean difference (WMD) and 95% confidence intervals (CIs) for the effect of berberine consumption on **(A)** TG (mg/dl); **(B)** TC (mg/dl); **(C)** LDL (mg/dl); **(D)** HDL (mg/dl); **(E)** FBG (mg/dl); **(F)** Insulin (mg/dl); **(G)** HbA1c (%); **(H)** HOMA-IR; **(I)** SBP (mmHg); **(J)** DBP (mmHg); **(K)** CRP (mg/L); **(L)** IL-6 (ng/L); **(M)** weight (kg); **(N)** BMI (kg/m^2^); **(O)** WC (cm); **(P)** ALT (U/L); and **(Q)** AST (U/L). TG, triglyceride; TC, total cholesterol; LDL, low-density lipoprotein; HDL, high-density lipoprotein; FBG, fasting blood glucose; HOMA-IR, homeostasis model assessment for insulin resistance; hemoglobin A1c, HbA1c; CRP, C-reactive protein; IL-6, interleukin 6; WC, waist circumference; ALT, alanine transaminase; AST, aspartate transaminase; SBP, systolic blood pressure; DBP, diastolic blood pressure; CI, confidence interval, weighted mean difference; WMD.

The characteristics of included studies are presented in Table 1. The WMD and 95% CI of TG (mg/dl), TC (mg/dl), LDL (mg/dl), HDL (mg/dl), FBG (mg/dl), insulin (mg/dl), HbA1c (%), HOMA-IR, SBP (mmHg), DBP (mmHg), CRP (mg/l), IL-6 (ng/l), weight (kg), BMI (kg/m^2^), WC (cm), ALT (U/L), AST (U/L), and their changes are presented in Figures 2A–Q respectively. The studies were published between 2004 and 2022 and were carried out in China (*n* = 22) (36, 39–43, 46, 47, 52, 53, 56, 59, 71–80), Iran (*n* = 19) (37, 38, 44, 45, 50, 51, 54, 57, 61, 63–65, 67, 69, 70, 81–83, 85), Italy (*n* = 2) (48, 84), Mexico (*n* = 2) (55, 62), India (*n* = 2) (60, 66), USA (*n* = 1) (49), and Pakistan (*n* = 1) (58). The study design of 48 studies were parallel (36–83) and one study was cross-over (84). In the intervention group, the mean age was between 25 and 65.5 years old, mean BMI was between 20.5 and 36.7 kg/m^2^. The BBR dose was between 200 ml and 6.25 g/d. The duration of intervention was between 4 and 104 weeks. The sample size in the intervention group was between 12 and 144. Four studies included only females (36, 38, 60, 72) and one study only included males (79) and the rest of the studies included both genders. Studies included participants with type 2 diabetes (43, 47–49, 54, 57, 58, 63, 65–67, 69–71, 76–78, 83), dyslipidemia (52, 53, 55, 79, 80), renal transplanted recipients (73), metabolic syndrome (39, 44, 62, 81, 82), polycystic ovary syndrome (36, 60, 72), acute coronary syndrome (59), non-alcoholic fatty liver disease (41, 50, 61, 64, 74, 75), acute ischemic stroke (46), women with benign breast disease (38), rheumatoid arthritis patients (37, 51), schizophrenia (56), hypertension (45), and healthy subjects (42, 84). Sample size in intervention and control group for SBP was 1,426 in total (intervention: 719, control: 707), DBP *n* = 1,426 (intervention: 719, control: 707), ALT *n* = 1 084 (intervention: 582, control: 502), AST *n* = 880 (intervention: 463, control: 417), body weight *n* = 1,706 (intervention: 879, control: 827), BMI *n* = 1,990 (intervention: 1,000, control: 990), WC *n* = 1,083 (intervention: 546, control: 537), FBG *n* = 2,713 (intervention: 1,377, control: 1,336), insulin *n* = 1,138 (intervention: 576, control: 562), HbA1c *n* = 1,566 (intervention: 822, control: 744), HOMA-IR *n* = 1,119 (intervention: 567, control: 552), CRP *n* = 662 (intervention: 326, control: 336), IL-6, *n* = 358 (intervention: 178, control: 180), TG *n* = 3,004 (intervention: 1,559, control: 1,445), TC *n* = 2,804 (intervention: 1,430, control: 1,374), LDL *n* = 2,824 (intervention: 1,457, control: 1,367), HDL *n* = 2,784 (intervention: 1,402, control: 1,346).

**Table 1:** Characteristic of included studies in the meta-analysis.

**Studies**	**Country**	**Study design**	**Participant**	**Sample size and sex**	**Sample size**		**Trial duration (week)**	**Means age**		**Means BMI**		**Intervention**		**Adverse effects**
					**IG**	**CG**		**IG**	**CG**	**IG**	**CG**	**Berberine (g/d)**	**Control group**	
Kong et al. (52)	China	R, DB, PC, parallel	Dyslipidemia	M/F: 43	32	11	12	NR	NR	NR	NR	1	Placebo	NA
Wu et al. (73)	China	R, PC, parallel	Renal transplanted recipients	M/F: 104	52	52	12	42.5 ± 10.8	39.6 ± 11.9	20.5 ± 3.4	20.4 ± 3.1	0.6	Control group	Constipation
Zhang et al. (78)	China	R, DB, PC, parallel	Type 2 diabetes	M/F: 110	58	52	12	51 ± 9	51 ± 10	25.2 ± 3.1	25.9 ± 3.8	1	Placebo	NA
Yin et al. (76)	China	R, PC, parallel	Type 2 diabetes	M/F: 31	15	16	12	25–75	25–75	26 ± 2.6	26 ± 2.4	1.5	Control group	Transient gastrointestinal adverse effects were reported
Kong et al. (63)	China	R, PC, parallel	Dyslipidemia	M/F: 39	23	16	8	NR	NR	NR	NR	1	Control group	No significant adverse effect was reported
Zhao et al. (80)	China	R, PC, parallel	Dyslipidemia	M/F: 51	35	16	12	43.6 ± 7.8	43.9 ± 8.9	NR	NR	1	Silymarin	NA
Ebrahimi-Mamaghani et al. (44)	Iran	R, PC, parallel	Metabolic Syndrome	M/F: 38	19	19	8	59.1 ± 12.2	53.8 ± 9	29.3 ± 3.3	31 ± 6.4	5	Placebo	NA
Golzarand et al. (83)	Iran	R, PC, parallel	Type 2 diabetes	M/F: 38	19	19	4	59.1 ± 12.2	53.8 ± 9	29.3 ± 3.3	31 ± 6.4	5	Placebo	NA
Gu et al. (47)	China	R, DB, PC, parallel	Type 2 diabetes	M/F: 60	30	30	12	51 ± 9	50 ± 10	25.1 ± 2.9	26.2 ± 3.6	1	Placebo	NA
Zhang et al. (77)	China	R, PC, parallel	Type 2 diabetes	M/F: 76	50	26	8	57 ± 8	56 ± 11	NR	NR	1	Metformin	No adverse effect was reported
Wei et al. (72)	China	R, PC, parallel	Polycystic Ovary syndrome	F: 59	31	28	12	25.74 ± 2.66	26.75 ± 2.62	25.57 ± 1.6	24.91 ± 1.66	1.5	Placebo	NA
Meng et al. (59)	China	R, PC, parallel	Acute coronary syndrome	M/F: 130	61	69	4	63.07 ± 10.41	63.28 ± 10.03	24.06 ± 2.49	23.5 ± 4.9	0.9	Control group	No sever adverse effect was reported
Shidfar et al. (67)	Iran	R, DB, PC, parallel	Type 2 diabetes	M/F: 42	21	21	12	53.1 ± 6.3	52.2 ± 4.9	27.3 ± 1	27.7 ± 1	3	Control group	NA
Yan et al. (74)	China	R, DB, PC, parallel	Nonalcoholic fatty liver disease	M/F: 124	62	62	16	50.69 ± 9.75	50.49 ± 10.72	28.08 ± 4.17	27.23 ± 2.8	1.5	Control group	NA
Derosa et al. (84)	Italy	R, DB, PC, crossover	Healthy subjects	M/F: 144	144	144	12	53 ± 11	53 ± 11	26.8 ± 2.1	26.8 ± 2.1	1	Placebo	No patients had serious adverse events in both groups; one patient reported headache and two patients reported transient flatulence
Cheng et al. (42)	China	R, PC, parallel	Healthy subjects	M/F: 23	12	11	4	53.75 ± 5.97	52.7 ± 4.55	22.56 ± 3.1	22.67 ± 1.91	1.2	Control group	NA
Pérez-Rubio et al. (62)	Mexico	R, DB, PC, parallel	Metabolic Syndrome	M/F: 24	12	12	12	38.1 ± 2.7	36.9 ± 3	36.1 ± 2.3	34.2 ± 3.6	1.5	Placebo	No significant adverse effect was reported
Kashkooli et al. (50)	Iran	R, PC, parallel	Nonalcoholic fatty liver disease	M/F: 80	40	40	12	43.2 ± 8.45	42.97 ± 8.56	NR	NR	0.75	Placebo	NA
An et al. (36)	China	R, DB, PC. parallel	Polycystic ovary syndrome	F: 87	44	43	12	28.2 ± 3.8	28.4 ± 4	24.6 ± 3.1	24.2 ± 3.2	1.5	Placebo	The commonly reported study side effects were nausea
Zilaee et al. (81)	Iran	R, DB, PC. parallel	Metabolic syndrome	M/F: 106	53	53	6	38.96 ± 9.04	40.89 ± 9.61	31.54 ± 3.92	32.37 ± 5.01	0.6	Placebo	NA
Fei-qi et al. (46)	China	R, PC, parallel	Acute ischemic stroke	M/F: 44	16	28	12	63.31 ± 8.1	66.25 ± 8.83	NR	NR	1.2	Control group	NA
Dai et al. (43)	China	R, PC, parallel	Type 2 diabetes	M/F: 69	36	33	104	55.31 ± 11.79	53.06 ± 10.36	24.5 ± 4.01	24.1 ± 4.36	0.3	Control group	NA
Yan et al. (75)	China	R, PC, parallel	Nonalcoholic fatty liver disease	M/F: 124	62	62	16	50.72 ± 9.76	50.64 ± 10.69	28.06 ± 4.17	27.27 ± 2.8	1.5	Control group	Adverse events were mild and mainly occurred in digestive system
Zilaee et al. (82)	Iran	R, DB, PC, parallel	Metabolic syndrome	M/F: 106	53	53	6	38.96 ± 9.04	40.89 ± 9.61	31.54 ± 3.92	32.37 ± 5.01	0.6	Placebo	NA
Kashkooli et al. (50)	Iran	R, PC, parallel	Nonalcoholic fatty liver disease	M/F: 80	40	40	12	43.65	42.97	NR	NR	0.75	Placebo	NA
Lazavi et al. (85)	Iran	R, PC, parallel	Type 2 diabetes	M/F: 42	21	21	8	57 ± 8	54 ± 7	29 ± 4	28 ± 3	200 ml	Control group	NA
Chang et al. (41)	China	R, PC, parallel	Nonalcoholic fatty liver disease	M/F: 80	41	39	16	51.2 ± 9.4	50.8 ± 10.4	27.4 ± 4.1	27.3 ± 3	1.5	Control group	NA
Guarino et al. (48)	Italy	R, DB, PC. parallel	Type 2 diabetes	M/F: 136	68	68	52	56 ± 8	55 ± 9	34 ± 4	34 ± 5	1	Placebo	NA
Mansouri et al. (57)	Iran	R, PC, parallel	Type 2 diabetes	M/F: 60	30	30	12	48.2 ± 4.3	48.2 ± 4.3	NR	NR	200 ml	Placebo	NA
Sharma et al. (66)	India	R, PC, parallel	Type 2 diabetes	M/F: 60	30	30	38	30–60	30–60	NR	NR	1.5	Conventional	No adverse effect was observed
Sharma et al. (66)	India	R, PC, parallel	Type 2 diabetes	M/F: 60	30	30	38	30–60	30–60	NR	NR	3	Conventional	NA
Asemani et al. (38)	Iran	R, TB, PC. parallel	Women with Benign Breast Disease	F: 85	44	41	8	36.17 ± 7.6	38.45 ± 6.9	NR	NR	480 ml	Placebo	No adverse effect was reported.
Rashidi et al. (63)	Iran	R, DB, PC, parallel	Type 2 diabetes	M/F: 84	42	42	4	50.18 ± 4.22	45.12 ± 9.55	29.81 ± 4.1	29.07 ± 5.07	1	Placebo	NA
Lazavi et al. (54)	Iran	R, PC, parallel	Type 2 diabetes	M/F: 46	23	23	8	56.86 ± 8.47	53.95 ± 6.57	29.22 ± 3.98	27.78 ± 3.45	200ml	Control group	No serious adverse effect were reported.
Tahmasebi et al. (70)	Iran	R, DB, PC, parallel	Type 2 diabetes	M/F: 80	40	40	6	54.05 ± 8	53.07 ± 7.74	NR	NR	1.5	Placebo	NA
Cao et al. (39)	China	R, PC, parallel	Metabolic syndrome	M/F: 80	40	40	4	65.5 ± 1.8	65.6 ± 1.8	NR	NR	1.2	Control group	Nausea and vomiting
Aryaeian et al. (37)	Iran	R, DB, PC, parallel	Rheumatoid Arthritis patients	M/F: 62	31	31	12	48.61 ± 11.69	47.1 ± 10.75	27.9 ± 6.06	29.46 ± 5.7	3	Placebo	NA
Sanjari et al. (65)	Iran	R, TB, PC, parallel	Type 2 diabetes	M/F: 80	42	38	12	51.8 ± 9.3	43.5 ± 10	27.2 ± 4.9	27.7 ± 5.3	0.48	Control group	No significant adverse effect was reported
Soltani et al. (69)	Iran	R, PC, parallel	Type 2 diabetes	M/F: 65	30	35	8	56.1 ± 7.2	57.6 ± 7.7	29.7 ± 4.4	29.5 ± 4.4	1	Control group	NA
Khorshidi-Sedehi et al. (51)	Iran	R, DB, PC, parallel	Rheumatoid arthritis patients	M/F: 62	31	31	12	48.61 ± 11.69	47.1 ± 10.75	27.9 ± 6.06	29.46 ± 5.7	1.5	Control group	NA
Li et al. (56)	China	R, DB, PC, parallel	Sschizophrenia	M/F: 49	27	22	8	44.74 ± 10.59	41.14 ± 11.51	24.73 ± 4.4	23.78 ± 2.6	0.9	Placebo	Abdominal distention, constipation, diarrhea, sinus bradycardia
Emamat et al. (45)	Iran	R, SB, PC, parallel	Hypertension	M/F: 84	42	42	8	53.62 ± 10.34	54.5 ± 10.13	28.21 ± 2.03	27.83 ± 2.32	10	Placebo	NA
León-Martínez et al. (55)	Mexico	R, DB, PC, parallel	Dyslipidemia	M/F: 24	12	12	12	46.8 ± 10.5	44.8 ± 9	29 ± 3.3	31.5 ± 4.3	1.5	Control group	NA
Memon et al. (58)	Pakistan	R, PC, parallel	Type 2 diabetes	M/F: 100	50	50	12	33.4 ± 2.96	33.26 ± 2.6	33.5 ± 2.53	34.7 ± 4.7	1.5	Metformin	NA
Zhao et al. (86)	China	R, DB, PC, parallel	Dyslipidemia	M: 84	42	42	12	49.5 ± 11.1	44.8 ± 13.5	26.3 ± 3.7	26.1 ± 3.8	1	Placebo	No significant adverse effect was reported
Harrison et al. (49)	USA	R, DB, PC. parallel	Type 2 diabetes	M/F: 66	33	17	18	58 ± 10.2	58 ± 10.7	36.7 ± 6.88	35 ± 6.18	1	Placebo	Diarrhea and abdominal discomfort
Harrison et al. (49)	USA	R, DB, PC. parallel	Type 2 diabetes	M/F: 67	34	16	18	53 ± 12.2	58 ± 10.7	36.3 ± 6.28	35 ± 6.18	2	Placebo	Diarrhea and abdominal discomfort
Chan et al. (40)	China	R, DB, PC. parallel	Schizophrenia	M/F: 113	58	55	12	39.3 ± 11.3	36.2 ± 10.8	29.3 ± 4.5	29.2 ± 4.2	0.6	Placebo	No serious adverse effect was reported
Wang et al. (71)	China	R, DB, PC. parallel	Type 2 diabetes	M/F: 175	84	91	12	52.07 ± 10.81	52.56 ± 9.44	25.78 ± 3.36	26.26 ± 3.42	1.2	Placebo	NA
Nejati et al. (61)	Iran	R, PC, parallel	Nonalcoholic fatty liver disease	M/F: 50	25	25	6	40.6 ± 8.8	42.2 ± 3.8	30.1 ± 4.1	29.9 ± 3.8	6.25	Control group	NA
Mishra et al. (60)	India	R, PC, parallel	Polycystic Ovary syndrome	F: 86	43	43	12	27.1 ± 5.1	27.67 ± 5.06	24.69 ± 2.99	25.46 ± 2.23	1	Metformin	NA

IG, intervention group; CG, control group; DB, double-blinded; SB, single-blinded; PC, placebo-controlled; CO, controlled; RA, randomized; NR, not reported; F, female; M, male; NR, not reported.

### Adverse effects

The adverse effect was reported in studies including Asemani et al. (38), Sharma et al. (66), Chan et al. (40), Meng et al. (59), Li et al. (56), Zhang et al. (78), Pérez-Rubio et al. (62), Zhao et al. (79), Sanjari et al. (65), Yin et al. (76), Kong et al. (53), Yan et al. (75), Lazavi et al. (54), Zhang et al. (77), An et al. (36), Cao et al. (39), Derosa et al. (84) and Harrison et al. (49). While no significant adverse effects were reported in some studies (38, 40, 53, 54, 59, 62, 65, 66, 77, 79), other studies reported mild to moderate gastrointestinal adverse effects including nausea, constipation, and diarrhea (36, 39, 49, 56, 73, 75, 76, 84).

### Qualitative data assessment

Based on the Cochrane Risk of Bias Assessment tool, a total of 38 studies were considered with a high risk of bias (36, 39, 41–50, 52–54, 57–66, 69, 71–77, 80, 82, 83), six studies had a moderate risk of bias (55, 67, 70, 78, 79, 84) and five studies had a low risk of bias (36–38, 51, 56) (Table 2).

**Table 2:** Risk of bias assessment.

**References**	**Random sequence generation**	**Allocation concealment**	**Selective reporting**	**Other sources of bias**	**Blinding (participants and personnel)**	**Blinding (outcome assessment)**	**Incomplete outcome data**	**General risk of bias**
Kong et al. (52)	U	H	L	H	H	H	L	High
Wu et al. (73)	U	H	L	H	H	H	L	High
Zhang et al. (78)	L	L	H	H	L	U	L	Moderate
Yin et al. (76)	U	H	H	H	H	H	H	High
Kong et al. (63)	U	H	L	H	H	H	L	High
Zhao et al. (80)	L	H	L	H	H	H	L	High
Ebrahimi-Mamaghani et al. (44)	L	L	H	H	H	H	L	High
Golzarand et al. (83)	L	H	H	L	H	H	L	High
Gu et al. (47)	L	H	H	H	L	U	L	High
Zhang et al. (77)	U	H	H	H	H	H	L	High
Wei et al. (72)	L	H	L	H	H	H	L	High
Meng et al. (59)	L	H	H	H	H	H	L	High
Shidfar et al. (67)	L	H	H	L	L	U	L	Moderate
Yan et al. (74)	L	H	H	H	L	U	L	High
Derosa et al. (84)	L	L	H	H	L	U	L	Moderate
Cheng et al. (42)	U	H	H	H	H	H	L	High
Pérez-Rubio et al. (62)	L	H	H	H	L	U	L	High
Kashkooli et al. (50)	L	H	H	H	H	H	L	High
An et al. (36)	L	H	H	H	L	U	L	High
Zilaee et al. (81)	L	L	L	H	L	U	L	Low
Fei-qi et al. (46)	L	H	L	H	H	H	L	High
Dai et al. (43)	L	H	H	H	H	H	L	High
Yan et al. (75)	L	H	L	H	H	H	H	High
Zilaee et al. (82)	L	H	H	H	L	U	L	High
Kashkooli et al. (50)	U	H	H	H	H	H	L	High
Lazavi et al. (85)	L	L	H	L	H	H	L	High
Chang et al. (41)	L	H	L	L	H	H	L	High
Guarino et al. (48)	L	H	H	H	L	U	L	High
Mansouri et al. (57)	L	H	H	H	H	H	L	High
Sharma et al. (66)	L	L	H	H	H	H	L	High
Asemani et al. (38)	L	L	H	L	L	L	L	Low
Rashidi et al. (63)	L	H	H	H	L	U	L	High
Lazavi et al. (54)	L	L	H	L	H	H	L	High
Tahmasebi et al. (70)	L	L	H	H	L	U	L	Moderate
Cao et al. (39)	U	H	H	H	H	H	L	High
Aryaeian et al. (37)	L	L	H	L	L	U	L	Low
Sanjari et al. (65)	L	H	H	H	L	L	L	High
Soltani et al. (69)	L	L	H	H	H	H	L	High
Khorshidi-Sedehi et al. (51)	L	L	L	L	L	U	L	Low
Li et al. (56)	L	L	L	H	L	U	L	Low
Emamat et al. (45)	L	L	H	H	H	H	L	High
León-Martínez et al. (55)	L	L	H	H	L	U	L	Moderate
Memon et al. (58)	U	H	H	H	H	H	L	High
Zhao et al. (86)	L	L	H	H	L	U	L	Moderate
Harrison et al. (49)	L	L	H	H	L	U	H	High
Chan et al. (40)	L	L	H	H	L	U	H	High
Wang et al. (71)	L	H	H	H	L	U	H	High
Nejati et al. (61)	L	L	H	H	H	H	L	High
Mishra et al. (60)	L	L	H	H	H	H	L	High

General low risk < 2 high risk.

General moderate risk = 2 high risk.

General high risk > 2 high risk.

### Effects of BBR supplementation on TG

A total of 38 effect sizes from 38 studies were included in the analysis of the effect of BBR supplementation on TG (Figure 2A) (36, 37, 39–42, 44, 47–50, 52–56, 58–63, 65–67, 71, 72, 74–81, 84). BBR significantly reduced TG compared to placebo (WMD = −23.70 mg/dl; 95% CI, −30.16 to −17.25; *P* < 0.001). The subgroup analysis showed that the effect of BBR on TG was significant in studies conducted on the baseline TG < 150 mg/dl (WMD = −18.18 mg/dl; 95% CI, −23.63 to −12.73; *P* < 0.001) and ≥150 mg/dl (WMD = −26.34 mg/dl; 95% CI, −33.78 to −18.90; *P* < 0.001), trial duration ≤ 8 weeks (WMD = −20.94; 95% CI, −30.70 to −11.17; *P* < 0.001) and trial duration >8 weeks (WMD = −25.59; 95% CI, −33.31 to −17.86; *P* < 0.001), intervention dose ≤ 1 g/d (WMD = −24.96 mg/dl; 95% CI, −38.79 to −11.13; *P* < 0.001) and >1 g/d (WMD = −24.89 mg/dl; 95% CI, −32.93 to −16.86; *P* < 0.001), overweight (25–29.9 kg/m^2^) (WMD = −26.88; 95% CI, −36.98 to −16.78; *P* < 0.001) and obese (>30 kg/m^2^) (WMD = −28.93; 95% CI, −44.38 to −13.48; *P* < 0.001), type 2 diabetes (WMD = −26.40; 95% CI, −33.91 to −18.89; *P* < 0.001), metabolic syndrome (WMD = −32.17; 95% CI, −59.74 to −4.60; *P* = 0.022), non-alcoholic fatty liver diseases (WMD = −32.30; 95% CI, −54.36 to −10.24; *P* = 0.004), and category of trails risk of bias, high risk of bias (WMD = −24.40; 95%CI −33.40 to −15.66; *P* < 0.001), moderate risk of bias (WMD = −27.55; 95%CI −38.65 to −16.46; *P* < 0.001) (Table 3). Between study heterogeneity was found for TG (I^2^ = 96.6%). The heterogeneity disappeared when subgroup analysis was performed on baseline TG (<150) (I^2^ = 25%, *P* = 0.238), and dyslipidemia (I^2^ = 20.2%, *P* = 0.286), low risk of bias (I^2^ = 0.0%, *P* = 0.668) (Table 3).

**Table 3:** Subgroup analyses of berberine supplementation on cardiovascular risk factors in adults.

	**No**	**WMD (95%CI)**	***P*** **-value**	**Heterogeneity**		
				***P*** **heterogeneity**	**I^2^**	***P*** **between sub-groups**
**Subgroup analyses of berberine on serum TG (mg/dl)**						
Overall effect	38	−23.70 (−30.16, −17.25)	< 0.001	< 0.001	96.6%	
Baseline TG (mg/dl)						
< 150	7	−18.18 (−23.63, −12.73)	< 0.001	< 0.238	25.0%	0.083
≥150	31	−26.34 (−33.78, −18.90)	< 0.001	< 0.001	97.1%	
Trial duration (week)						
≤ 8	11	−20.94 (−30.70, −11.17)	< 0.001	0.022	52.0%	0.464
>8	27	−25.59 (−33.31, −17.86)	< 0.001	< 0.001	97.5%	
Intervention dose (g/day)						
≤ 1	18	−24.96 (−38.79, −11.13)	< 0.001	< 0.001	91.5%	0.994
>1	20	−24.89 (−32.93, −16.86)	< 0.001	< 0.001	97.8%	
Baseline BMI (kg/m^2^)						
Normal (18.5–24.9)	5	−4.47 (−35.76, −26.82)	0.779	< 0.001	96.5%	0.371
Overweight (25–29.9)	18	−26.88 (−36.98, −16.78)	< 0.001	< 0.001	96.6%	
Obese (>30)	7	−28.93 (−44.38, −13.48)	< 0.001	< 0.001	80.9%	
Health status						
Dyslipidemia	5	−26.53 (−47.04, −6.02)	0.011	0.286	20.2%	< 0.001
Type 2 diabetes	15	−26.40 (−33.91, −18.89)	< 0.001	< 0.001	94.7%	
Metabolic syndrome	4	−32.17 (−59.74, −4.60)	0.022	< 0.001	75.9%	
Nonalcoholic fatty liver disease	5	−32.30 (−54.36, −10.24)	0.004	< 0.001	86.5%	
Others	9	−8.41 (−22.52, 5.68)	0.242	< 0.001	93.8%	
Risk of bias						
High	30	−24.40 (−33.14, −15.66)	< 0.001	< 0.001	97.2%	0.102
Moderate	5	−27.55 (−38.65, −16.46)	< 0.001	< 0.001	90.0%	
Low	3	−8.79 (−22.98, 5.40)	0.225	0.668	0.0%	
**Subgroup analyses of berberine on serum TC (mg/dl)**						
Overall effect	34	−20.64 (−23.65, −17.63)	< 0.001	< 0.001	85.4%	
Baseline TC (mg/dl)						
< 200	10	−12.10 (−18.86, −5.34)	< 0.001	< 0.001	78.0%	0.003
≥200	24	−23.81 (−27.55, 20.06)	0.035	< 0.001	86.2%	
Trial duration (week)						
≤ 8	10	−18.09 (−26.21, −9.97)	< 0.001	< 0.001	77.7%	0.475
>8	24	−21.30 (−24.74, 17.86)	< 0.001	< 0.001	87.3%	
Intervention dose (g/day)						
≤ 1	16	−21.30 (−28.23, −14.36)	< 0.001	< 0.001	90.7%	0.918
>1	18	−20.90 (−23.87, −17.93)	< 0.001	< 0.001	73.9%	
Baseline BMI (kg/m^2^)						
Normal (18.5–24.9)	5	−10.58 (−30.39, 9.23)	0.295	< 0.001	95.3%	0.630
Overweight (25–29.9)	18	−20.42 (−23.52, −17.31)	< 0.001	< 0.001	72.8%	
Obese (>30)	4	−20.20 (−30.23, −10.16)	< 0.001	0.005	77.0%	
Health status						
Dyslipidemia	5	−35.00 (−56.05, −13.94)	0.001	< 0.001	86.8%	< 0.001
Type 2 diabetes	12	−22.20 (−26.87, −17.54)	< 0.001	< 0.001	78.0%	
Metabolic syndrome	3	−26.85 (−29.47, −24.22)	< 0.001	0.807	0.00%	
Nonalcoholic fatty liver disease	5	−18.24 (−24.71, −11.78)	< 0.001	0.017	66.9%	
Others	9	−13.10(−22.05, −4.15)	0.004	< 0.001	92.0%	
Risk of bias						
High	26	−20.59 (−24.59, −16.58)	< 0.001	< 0.001	87.5%	0.265
Moderate	5	−24.07 (−28.25, −19.88)	< 0.001	0.019	66.2%	
Low	3	−13.25 (−28.64, 2.13)	0.091	0.013	77.1%	
**Subgroup analyses of berberine on serum LDL (mg/dl)**						
Overall effect	35	−9.63 (−13.87, −5.39)	< 0.001	< 0.001	96.1%	
Baseline LDL (mg/dl)						
< 100	4	−3.31 (−13.33, 6.69)	0.516	0.075	56.6%	0.209
≥100	31	−10.34 (−14.82, −5.86)	< 0.001	< 0.001	96.5%	
Trial duration (week)						
≤ 8	9	−11.78 (−17.74, −5.81)	< 0.001	0.006	62.7%	0.450
>8	26	−8.79 (−13.74, −3.84)	< 0.001	< 0.001	97.1%	
Intervention dose (g/day)						
≤ 1	17	−13.15 (−19.36, −6.94)	< 0.001	< 0.001	92.3%	0.099
>1	18	−6.39 (−11.47, −1.30)	0.014	< 0.001	95.6%	
Baseline BMI (kg/m^2^)						
Normal (18.5–24.9)	5	−6.76 (−20.53, 6.99)	0.335	< 0.001	95.1%	0.454
Overweight (25–29.9)	18	−13.15 (−18.75, −7.55)	< 0.001	< 0.001	95.5%	
Obese (>30)	6	1.11 (−26.48, 28.70)	0.937	< 0.001	98.4%	
Health status						
Dyslipidemia	5	−17.92 (−28.35, −7.48)	0.001	0.065	54.9%	0.001
Type 2 diabetes	14	−5.42 (−12.79, 1.95)	0.150	< 0.001	96.7%	
Metabolic Syndrome	2	−22.30 (−30.90, −13.71)	< 0.001	0.348	0.00%	
Nonalcoholic fatty liver disease	5	– 6.50 (−7.72, −5.29)	< 0.001	0.883	0.00%	
Others	9	−11.69 (−21.17, −2.20)	0.016	< 0.001	95.6%	
Risk of bias						
High	27	−7.20 (−11.51, −2.89)	0.001	< 0.001	94.2%	0.004
Moderate	5	−19.20 (−24.90, −13.50)	< 0.001	< 0.001	85.6%	
Low	3	−14.55 (−22.47, −6.64)	< 0.001	0.187	40.4%	
**Subgroup analyses of berberine on serum HDL (mg/dl)**						
Overall effect	34	1.37 (0.41, 2.33)	0.005	< 0.001	92.7%	
Baseline HDL (mg/dl)						
< 40	8	1.17 (0.08, 2.27)	0.035	< 0.001	89.6%	0.960
≥40	26	1.22 (−0.18, 2.63)	0.088	< 0.001	90.6%	
Trial duration (week)						
≤ 8	9	2.17 (0.10, 4.23)	0.039	< 0.001	77.3%	0.371
>8	25	1.10 (−0.02, 2.22)	0.055	< 0.001	94.2%	
Intervention dose (g/day)						
≤ 1	16	0.49 (−1.86, 2.85)	0.682	< 0.001	92.2%	0.307
>1	18	1.81 (0.88, 2.75)	< 0.001	< 0.001	90.1%	
Baseline BMI (kg/m^2^)						
Normal (18.5–24.9)	5	−1.34 (−6.07, 3.39)	0.579	< 0.001	94.9%	0.048
Overweight (25–29.9)	18	0.91 (0.04, 1.78)	0.039	< 0.001	78.3%	
Obese (>30)	5	4.85 (1.52, 8.17)	0.004	< 0.001	88.5%	
Health status						
Dyslipidemia	5	−1.96 (−6.85, 2.92)	0.430	0.104	47.9%	0.004
Type 2 diabetes	14	1.65 (0.19, 3.10)	0.026	< 0.001	93.4%	
Metabolic Syndrome	2	6.90 (2.42, 11.37)	0.002	0.078	60.8%	
Nonalcoholic fatty liver disease	5	−0.00 (−0.22, 0.20)	0.957	0.988	0.00%	
Others	9	1.03 (−1.26, 3.34)	0.377	< 0.001	91.3%	
Risk of bias						
High	26	1.22 (0.08, 2.36)	0.035	< 0.001	93.9%	0.106
Moderate	5	0.30 (−1.35, 1.97)	0.718	0.009	70.6%	
Low	3	5.46 (0.93, 9.99)	0.018	0.042	68.5%	
**Subgroup analyses of berberine on serum FBG (mg/dl)**						
Overall effect	35	−7.74 (−10.79, −4.70)	< 0.001	< 0.001	97.0%	
Baseline FBG (mg/dl)						
< 100	10	−1.81 (−4.22, 0.59)	0.139	< 0.001	82.9%	0.003
≥100	25	−10.61 (−15.94, −5.27)	< 0.001	< 0.001	97.8%	
Trial duration (week)						
≤ 8	14	−2.43 (−8.68, 3.81)	0.446	< 0.001	94.0%	0.026
>8	21	−10.83 (−14.73, −6.92)	< 0.001	< 0.001	97.8%	
Intervention dose (g/day)						
≤ 1	14	−4.73 (−8.75, −0.71)	0.021	< 0.001	89.5%	0.116
>1	21	−9.88 (−14.88, −4.88)	< 0.001	< 0.001	98.0%	
Baseline BMI (kg/m^2^)						
Normal (18.5–24.9)	6	−3.44 (−5.75, −1.13)	0.003	0.089	47.6%	0.003
Overweight (25–29.9)	18	−9.21 (−12.90, −5.52)	< 0.001	< 0.001	96.4%	
Obese (>30)	4	−0.17 (−3.96, 3.62)	0.930	0.322	14.0%	
Health status						
Dyslipidemia	1	−3.60 (−8.81, 1.61)	0.176	< 0.001	–	0.002
Type 2 diabetes	15	−16.84 (−24.51, −9.17)	< 0.001	< 0.001	94.5%	
Metabolic syndrome	4	6.85 (−2.46, 16.16)	0.150	< 0.001	89.0%	
Nonalcoholic fatty liver disease	5	−2.21 (−4.41, −0.02)	0.048	0.004	73.9%	
Others	10	−2.80 (−5.92, 0.32)	0.079	< 0.001	86.9%	
Risk of bias						
High	27	−6.76 (−10.61, −2.90)	0.001	< 0.001	96.6%	0.623
Moderate	5	−13.56 (−26.81, −0.31)	0.045	< 0.001	98.7%	
Low	3	−6.58 (−19.31, 6.14)	0.311	< 0.001	94.6%	
**Subgroup analyses of berberine on serum Insulin (mg/dl)**						
Overall effect	16	−3.27 (−4.46, −2.07)	< 0.001	< 0.001	95.3%	
Trial duration (week)						
≤ 8	6	−3.74 (−6.45, −1.04)	0.007	< 0.001	96.2%	0.777
>8	10	−3.28 (−5.01, −1.54)	< 0.001	< 0.001	93.7%	
Intervention dose (g/day)						
≤ 1	6	−2.54 (−5.01, −0.06)	0.044	< 0.001	95.4%	0.367
>1	10	−3.91 (−5.58, −2.24)	< 0.001	< 0.001	95.4%	
Baseline BMI (kg/m^2^)						
Normal (18.5–24.9)	3	−2.74 (−7.26, 1.78)	0.235	< 0.001	97.1%	0.626
Overweight (25–29.9)	11	−4.11 (−5.87, −2.35)	< 0.001	< 0.001	90.9%	
Obese (>30)	1	−2.98 (−4.66, −1.29)	0.001	–	–	
Health status						
Type 2 diabetes	8	−3.35 (−4.98, −1.72)	< 0.001	< 0.001	87.3%	0.502
Metabolic syndrome	1	−7.30 (−16.96, 2.36)	0.139	–	–	
Nonalcoholic fatty liver disease	2	−6.09 (−16.74, 4.54)	0.261	< 0.001	98.1%	
Others	5	−2.08 (−3.74, −0.42)	0.014	< 0.001	94.6%	
Risk of bias						
High	12	−4.34 (−6.50, −2.17)	< 0.001	< 0.001	94.8%	0.078
Moderate	2	−1.90 (−2.42, −1.38)	< 0.001	0.928	0.0%	
Low	2	−1.15 (−3.57, 1.25)	0.346	0.010	85.0%	
**Subgroup analyses of berberine on serum HbA1c (%)**						
Overall effect	21	−0.45 (−0.68, −0.23)	< 0.001	< 0.001	92.5%	
Trial duration (week)						
≤ 8	5	0.12 (−0.47, 0.73)	0.680	< 0.001	83.2%	0.027
>8	16	−0.61 (−0.85, −0.22)	< 0.001	< 0.001	93.2%	
Intervention dose (g/day)						
≤ 1	10	−0.21 (−0.67, 0.25)	0.374	< 0.001	94.3%	0.111
>1	11	−0.64 (−0.92, −0.37)	< 0.001	< 0.001	87.4%	
Baseline BMI (kg/m^2^)						
Normal (18.5–24.9)	2	0.53 (0.28, 0.79)	< 0.001	0.909	0.0%	< 0.001
Overweight (25–29.9)	13	−0.41 (−0.53, −0.29)	< 0.001	0.057	41.6%	
Obese (>30)	4	−0.94 (−1.36, −0.53)	< 0.001	0.003	78.8%	
Health status						
Type 2 diabetes	15	−0.51 (−0.870, −0.16)	0.004	< 0.001	91.7%	0.658
Non-alcoholic fatty liver disease	3	−0.34 (−0.460, −0.22)	< 0.001	0.180	41.7%	
Others	3	−0.29 (−1.592, 1.00)	0.660	< 0.001	92.8%	
Risk of bias						
High	18	−0.52 (−0.77, −0.27)	< 0.001	< 0.001	92.8%	< 0.001
Moderate	2	−0.39 (−0.88, 0.09)	0.112	0.082	66.9%	
**Subgroup analyses of berberine on HOMA-IR**						
Overall effect	14	−1.04 (−1.55, −0.52)	< 0.001	< 0.001	99.1%	
Trial duration (week)						
≤ 8	5	−0.78 (−1.69, 0.12)	0.091	< 0.001	90.5%	0.466
>8	9	−1.13 (−1.40, −0.86)	< 0.001	< 0.001	87.0%	
Intervention dose (g/day)						
≤ 1	6	−1.37 (−2.12, −0.62)	< 0.001	< 0.001	90.5%	0.217
>1	8	−0.77 (−1.36, −0.18)	0.010	< 0.001	99.0%	
Baseline BMI (kg/m^2^)						
Normal (18.5–24.9)	2	−0.93 (−1.73,−0.14)	0.021	0.059	71.9%	0.683
Overweight (25–29.9)	9	−1.03 (−1.50, −0.56)	< 0.001	< 0.001	76.8%	
Obese (>30)	2	−1.31 (−1.90, −0.73)	< 0.001	< 0.001	92.4%	
Health status						
Type 2 diabetes	8	−1.25 (−1.62, −0.88)	< 0.001	< 0.001	92.8%	0.152
Metabolic syndrome	1	0.40 (−4.70, 5.50)	0.878	–	–	
Nonalcoholic fatty liver disease	2	−0.68 (−1.12, −0.23)	0.003	0.518	0.0%	
Others	5	−0.62 (−1.24, −0.00)	0.047	< 0.001	87.6%	
Risk of bias						
High	10	−1.12 (−1.59, −0.65)	< 0.001	< 0.001	85.6%	0.011
Moderate	2	−1.10 (−1.18, −1.02)	< 0.001	0.498	0.0%	
Low	2	−0.25 (−0.80, 0.30)	0.374	0.012	84.2%	
**Subgroup analyses of berberine on SBP (mmHg)**						
Overall effect	20	−5.46 (−8.17, −2.76)	< 0.001	< 0.001	86.3 %	
Baseline SBP (mmHg)						
< 120	13	−2.93 (−4.09, −1.76)	< 0.001	0.480	0.0%	0.028
≥120	7	−10.29 (−16.75, −3.82)	0.002	< 0.001	91.7%	
Trial duration (week)						
≤ 8	8	−6.83 (−11.98, −1.68)	0.009	< 0.001	85.2%	0.491
>8	12	−4.68 (−7.99, −1.36)	0.006	< 0.001	87.9%	
Intervention dose (g/day)						
≤ 1	11	−3.85 (−7.50, −0.19)	0.039	< 0.001	88.9%	0.190
>1	9	−7.58 (−11.79, −3.36)	< 0.001	< 0.001	82.8%	
Baseline BMI (kg/m^2^)						
Normal (18.5–24.9)	2	−2.12 (−5.52, 1.28)	0.223	0.363	0.0%	0.089
Overweight (25–29.9)	12	−5.20 (−8.48, −1.92)	0.002	< 0.001	79.5%	
Obese (>30)	4	−9.69 (−15.77, −3.60)	0.002	< 0.001	90.1%	
Health status						
Dyslipidemia	2	−1.33 (−4.64, 1.97)	0.428	0.779	0.0%	0.034
Type 2 diabetes	12	−6.99 (−11.29, −2.68)	0.001	< 0.001	89.9%	
Metabolic syndrome	2	−5.70 (−8.49, −2.91)	< 0.001	0.839	0.0%	
Non-alcoholic fatty liver disease	1	−0.85 (−3.50, 1.80)	0.530	–	–	
Others	3	−3.76 (−6.97, −0.55)	0.022	0.281	21.1%	
Risk of bias						
High	15	−6.73 (−10.19, −3.27)	< 0.001	< 0.001	88.4%	0.057
Moderate	4	−2.27 (−4.33, −0.21)	0.030	0.667	0.0%	
**Subgroup analyses of berberine on DBP (mmHg)**						
Overall effect	20	−2.74 (−5.63, 0.15)	0.063	< 0.001	94.9%	
Baseline DBP (mmHg)						
< 80	9	−0.85 (−3.44, 1.72)	0.516	< 0.001	84.1%	0.193
≥80	11	−4.20 (−8.52, 0.12)	0.057	< 0.001	95.9%	
Trial duration (week)						
≤ 8	8	−3.12 (−5.47, −0.77)	0.009	0.002	70.0%	0.811
>8	12	−2.52 (−6.88, 1.84)	0.257	< 0.001	96.8%	
Intervention dose (g/day)						
≤ 1	11	−2.46 (−6.86, 1.93)	0.273	< 0.001	97.2%	0.840
>1	9	−2.95 (−4.90, −1.00)	0.003	0.037	51.2%	
Baseline BMI (kg/m^2^)						
Normal (18.5–24.9)	2	−1.24 (−3.31, 0.82)	0.237	0.628	0.0%	0.359
Overweight (25–29.9)	12	−1.61 (−4.10, 0.87)	0.204	< 0.001	84.2%	
Obese (>30)	4	−7.40 (−15.58, 0.76)	0.076	< 0.001	97.5%	
Health status						
Dyslipidemia	2	−1.66 (−4.76, 1.42)	0.290	0.276	15.7%	0.002
Type 2 diabetes	12	−2.70 (−7.38, 1.98)	0.258	< 0.001	96.7%	
Metabolic Syndrome	2	−5.18 (−6.91, −3.45)	< 0.001	0.502	0.0%	
Nonalcoholic fatty liver disease	1	0.13 (−1.72, 1.98)	0.891	–	–	
Others	3	−2.88 (−8.67, 2.90)	0.328	0.001	86.0%	
Risk of bias						
High	15	−3.30 (−7.01, 0.39)	0.080	< 0.001	96.0%	0.203
Moderate	4	−1.21 (−2.70, 0.26)	0.108	0.734	0.0%	
**Subgroup analyses of berberine on serum CRP (mg/l)**						
Overall effect	9	0.05 (−0.59, 0.68)	0.887	< 0.001	97.4%	
Trial duration (week)						
≤ 8	5	0.53 (−0.45, 1.51)	0.290	< 0.001	97.6%	0.044
>8	4	−1.19 (−2.55, 0.16)	0.085	0.034	65.4%	
Intervention dose (g/day)						
≤ 1	4	−0.56 (−0.87, −0.25)	< 0.001	0.004	77.4%	0.391
>1	5	0.24 (−1.59, 2.08)	0.791	< 0.001	97.0%	
Baseline BMI (kg/m^2^)						
Normal (18.5–24.9)	3	−0.26 (−0.73, 0.20)	0.269	< 0.001	96.9%	0.151
Overweight (25–29.9)	3	−1.47 (−4.23, 1.27)	0.293	< 0.001	87.3%	
Obese (>30)	1	−1.06(−1.77, −0.34)	0.003	–	–	
Health status						
Type 2 diabetes	3	−0.26 (−1.31, 0.78)	0.621	0.003	82.5%	0.838
Metabolic Syndrome	2	0.97 (−3.00, 4.95)	0.630	< 0.001	98.8%	
Others	4	−0.15 (−0.65, 0.33)	0.531	0.002	79.8%	
Risk of bias						
High	6	0.51 (−0.21, 1.24)	0.167	< 0.001	98.3%	0.053
Low	2	−4.29 (−11.56, 2.97)	0.247	0.008	85.9%	
**Subgroup analyses of berberine on serum IL-6 (ng/l)**						
Overall effect	4	−0.53 (−1.11, 0.05)	0.073	< 0.001	94.7%	
Trial duration (week)						
≤ 8	3	−0.56 (−1.21, 0.08)	0.087	< 0.001	96.4%	0.790
>8	1	−0.40 (−1.43, 0.63)	0.448	–	–	
Intervention dose (g/day)						
≤ 1	2	−0.55 (−0.74, −0.36)	< 0.001	0.766	0.0%	0.634
>1	2	−1.21 (−3.93, 1.50)	0.380	< 0.001	92.5%	
Baseline BMI (kg/m^2^)						
Normal (18.5–24.9)	1	−0.56 (−0.75, −0.37)	< 0.001	–	–	0.422
Overweight (25–29.9)	2	−1.49 (−3.75, 0.77)	0.196	0.013	83.9%	
**Subgroup analyses of berberine on weight (kg)**						
Overall effect	21	−0.84 (−1.34, −0.34)	< 0.001	0.187	21.2%	
Trial duration (week)						
≤ 8	4	−0.86 (−2.84, 1.11)	0.393	0.687	0.0%	0.987
>8	17	−0.87 (−1.44, −0.31)	0.002	0.092	33.1%	
Intervention dose (g/day)						
≤ 1	9	−0.51 (−1.09, 0.06)	0.079	0.219	25.3%	0.059
>1	12	−1.52 (−2.40, −0.65)	0.001	0.349	9.8%	
Baseline BMI (kg/m^2^)						
Normal (18.5–24.9)	1	0.15 (−1.14, 1.44)	0.820	–	0.0%	0.200
Overweight (25–29.9)	14	−0.83 (−1.19, −0.47)	< 0.001	0.458	62.1%	
Obese (>30)	4	−1.90 (−3.94, −0.14)	0.068	0.048	28.2%	
Health status						
Dyslipidemia	1	−1.40 (−7.73, 4.93)	0.665	–	–	0.131
Type 2 diabetes	9	−1.58 (−2.52, −0.64)	0.001	0.391	5.3%	
Nonalcoholic fatty liver disease	5	−1.63 (−2.97, −0.29)	0.017	0.837	0.0%	
Others	6	−0.28 (−1.05, 0.49)	0.478	0.053	54.1%	
Risk of bias						
High	16	−1.02 (−1.53, −0.50)	< 0.001	0.312	12.3%	0.092
Moderate	4	0.07 (−0.76, 0.91)	0.862	0.439	0.0%	
**Subgroup analyses of berberine on BMI (kg/m** ^**2**^ **)**						
Overall effect	24	−0.25 (−0.46, −0.04)	0.020	0.010	44.7%	
Trial duration (week)						
≤ 8	8	−0.18 (−0.57, 0.21)	0.367	0.765	0.0%	0.713
>8	16	−0.26 (−0.52, −0.01)	0.041	0.001	59.8%	
Intervention dose (g/day)						
≤ 1	10	−0.20 (−0.53, 0.13)	0.241	0.002	66.3%	0.674
>1	14	−0.29 (−0.55, −0.03)	0.027	0.316	12.6%	
Baseline BMI (kg/m^2^)						
Normal (18.5–24.9)	2	−0.07 (−1.66, 1.52)	0.931	0.002	89.1%	0.969
Overweight (25–29.9)	17	−0.27 (−0.39, −0.15)	< 0.001	0.504	0.0%	
Obese (>30)	5	−0.25 (−1.13, 0.80)	0.637	0.003	99.6%	
Health status						
Dyslipidemia	2	−0.44 (−1.34, 0.45)	0.334	0.577	0.0%	0.733
Type 2 diabetes	9	−0.35 (−0.84, 0.12)	0.149	0.033	52.1%	
Metabolic syndrome	3	−0.41 (−1.24, 0.41)	0.325	0.446	0.00%	
Nonalcoholic fatty liver disease	3	−0.52 (−1.16, 0.11)	0.106	0.454	0.00%	
Others	7	−0.10 (−0.42, 0.20)	0.495	0.003	69.8%	
Risk of bias						
High	18	−0.28 (−0.57, 0.01)	0.058	0.008	50.3%	0.585
Moderate	5	−0.09 (−0.29, 0.10)	0.361	0.449	0.0%	
**Subgroup analyses of berberine on WC (cm)**						
Overall effect	11	−1.77 (−3.55, 0.01)	0.051	< 0.001	92.9%	
Intervention dose (g/day)						
≤ 1	4	−1.02 (−3.99, 1.94)	0.499	< 0.001	97.1%	0.279
>1	7	−2.75 (−3.72, −1.77)	< 0.001	0.825	0.0%	
Baseline BMI (kg/m^2^)						
Normal (18.5–24.9)	2	0.64 (−6.24, 7.53)	0.854	< 0.001	97.3%	0.481
Overweight (25–29.9)	7	−1.37 (−2.71, −0.03)	0.044	< 0.001	77.8%	
Obese (>30)	2	−5.37 (−12.72, 1.96)	0.151	< 0.001	92.2%	
Risk of bias						
High	8	−2.26 (−4.99, 0.45)	0.103	< 0.001	94.5%	0.104
Moderate	2	0.39 (−0.03, 0.83)	0.073	0.912	0.0%	
**Subgroup analyses of berberine on ALT (U/L)**						
Overall effect	12	−4.22 (−8.75, 0.31)	0.068	< 0.001	92.3%	
Trial duration (week)						
≤ 8	2	−0.53 (−2.57, 1.50)	0.606	0.433	0.0%	0.148
>8	10	−5.34 (−11.53, 0.84)	0.090	< 0.001	93.7%	
Intervention dose (g/day)						
≤ 1	8	−4.09 (−9.67, 1.49)	0.151	< 0.001	94.9%	0.997
>1	4	−4.07 (−10.81, 2.67)	0.237	0.121	48.4%	
Baseline BMI (kg/m^2^)						
Normal (18.5–24.9)	1	−4.70 (−11.24, 1.84)	0.159	–	–	0.289
Overweight (25–29.9)	5	0.19 (−1.40, 1.79)	0.811	0.220	30.2%	
Obese (>30)	3	−3.86 (−14.71, 6.99)	0.485	0.066	63.2%	
Health status						
Dyslipidemia	2	−4.42 (−36.17, 27.32)	0.785	< 0.001	95.3%	0.859
Type 2 diabetes	4	−1.60 (−5.27, 2.06)	0.393	0.155	42.8%	
Nonalcoholic fatty liver disease	4	−5.89 (−16.16, 4.38)	0.261	< 0.001	90.4%	
Others	2	−0.73 (−6.57, 5.09)	0.805	0.069	69.8%	
**Subgroup analyses of berberine on AST(U/L)**						
Overall effect	9	−2.94(−8.68, 2.81)	0.316	< 0.001	95.8%	
Trial duration (week)						
≤ 8	2	−0.33 (−2.09, 1.42)	0.709	0.758	0.0%	0.493
>8	7	−3.45 (−12.21, 5.30)	0.439	< 0.001	96.8%	
Intervention dose (g/day)						
≤ 1	6	−3.51 (−12.14, 5.12)	0.425	< 0.001	97.4%	0.653
>1	3	−1.46 (−3.74, 0.82)	0.210	0.910	0.0%	
Baseline BMI (kg/m^2^)						
Overweight (25–29.9)	5	0.24 (−1.83, 2.31)	0.821	0.063	55.3%	0.620
Obese (>30)	1	−0.90 (−4.90, 3.10)	0.660	–	–	
Health status						
Dyslipidemia	2	0.53 (−24.44, 25.52)	0.966	0.002	89.9%	0.084
Type 2 diabetes	2	−0.20 (−2.14, 1.74)	0.839	0.992	0.0%	
Nonalcoholic fatty liver disease	4	−6.27 (−16.78, 4.22)	0.242	< 0.001	96.9%	
Others	1	2.50 (0.97, 4.03)	0.001	–	–	

ALT, alanine transaminase; AST, aspartate transaminase; BMI, body mass index; CI, confidence interval; CRP, c-reactive protein; FBG, fasting blood glucose; HbA1c, hemoglobin A1c; HDL, high-density lipoprotein; HOMA-IR, homeostatic model assessment for insulin resistance; LDL, low-density lipoprotein; DBP, diastolic blood pressure; SBP, systolic blood pressure; TC, total cholesterol, TG, triglyceride; WC, waist circumference; WMD, weighted mean differences; IL-6, Interleukin 6.

### Effects of BBR supplementation on TC

A total of 34 effect sizes from 28 studies were included in the meta-analysis of the effect of BBR supplementation on TC (Figure 2B) (36, 37, 39–42, 44, 47, 48, 50, 52–56, 58–61, 63, 65–67, 71, 72, 74–76, 78–81, 84). BBR significantly reduced TC compared to placebo (WMD = −20.64 mg/dl; 95%CI, −23.65 to −17.63; *P* < 0.001). The subgroup analysis showed that the effect of BBR supplementation on TC was significant in studies conducted on the baseline TC < 200 mg/dl (WMD = −12.10 mg/dl; 95%CI, −18.86 to −5.34; *P* < 0.001), ≥200 mg/dl (WMD = −23.81 mg/dl; 95%CI, −27.55 to 20.06; *P* = 0.035), trial duration ≤ 8 weeks (WMD = −18.09; 95%CI, −26.21 to −9.97; *P* < 0.001) and >8 weeks (WMD = −21.30; 95%CI, −24.74 to 17.86; *P* < 0.001), intervention dose ≤ 1 g/d (WMD = −21.30 g/d; 95%CI, −28.23 to −14.36; *P* < 0.001) and > 1 g/d (WMD = −20.90 g/d; 95%CI, −23.87 to −17.93; *P* < 0.001), overweight (25–29.9 kg/m^2^) (WMD = −20.42; 95%CI, −23.52 to −17.31; *P* < 0.001), obese (>30 kg/m^2^) (WMD = −20.20 mg/dl; 95%CI, −30.23 to −10.16; *P* < 0.001), dyslipidemia (WMD = −35.00; 95%CI, −56.05 to −13.94; *P* = 0.001), type 2 diabetes (WMD = −22.20; 95%CI, −26.87 to −17.54; *P* < 0.001), metabolic syndrome (WMD = −20.85; 95%CI, −29.47 to −24.22; *P* < 0.001), non-alcoholic fatty liver diseases (WMD = −18.24; 95%CI, −24.71 to −11.78; *P* < 0.001), other health status (WMD = −13.10; 95%CI, −22.05 to −4.15; *P* = 0.004), and category of risk of bias of trails, high risk of bias (WMD = −20.59; 95%CI −24.59 to −16.58; *P* < 0.001), moderate risk of bias (WMD = −24.07; 95%CI −28.25 to −19.88; *P* < 0.001) (Table 3). Between study heterogeneity was found for TC (I^2^ = 85.4%). The heterogeneity diminished when subgroup analysis was conducted on health status (metabolic syndrome) (I^2^ = 0.00%, *P* = 0.807) (Table 3).

### Effect of BBR supplementation on LDL

A total of 35 effect sizes from 35 studies were included in the meta-analysis of the effect of BBR supplementation on LDL (Figure 2C) (36, 37, 40–42, 44, 47–50, 52–56, 58–61, 63, 65–67, 71, 72, 74–76, 78–81, 84). BBR significantly reduced LDL compared to placebo (WMD = −9.63 mg/dl; 95%CI, −13.87 to −5.39; *P* < 0.001). The subgroup analysis showed that the effect of BBR supplementation on LDL was significant in studies conducted on the baseline LDL ≥ 100 mg/dl (WMD = −10.34 mg/dl; 95%CI, −14.82 to −5.86; *P* < 0.001), trial duration ≤ 8 weeks (WMD = −11.78; 95%CI, −17.74 to −5.81; *P* < 0.001), trial duration > 8 weeks (WMD = −8.79; 95%CI, −13.74 to −3.84; *P* < 0.001), intervention dose ≤ 1 g/d (WMD = −13.15 g/dl; 95%CI, −19.36 to −6.94; *P* < 0.001), and >1 g/d (WMD = −6.39; 95%CI, −11.47 to −1.30; *P* = 0.014), overweight (25–29.9 kg/m^2^) (WMD = −13.15; 95%CI, −18.75 to −7.55; *P* < 0.001), dyslipidemia (WMD = −17.92; 95%CI, −28.35 to −7.48; *P* = 0.001), metabolic syndrome (WMD = −22.30; 95%CI, −30.90 to −13.71; *P* < 0.001), non-alcoholic fatty liver disease (WMD = −6.50; 95%CI, −7.72 to −5.29; *P* < 0.001), other health status (WMD = −11.69; 95%CI, −21.17 to −2.20; *P* = 0.016), and category of risk of bias of trails, high risk of bias (WMD = −7.20; 95%CI −11.51 to −2.89; *P* = 0.001), moderate risk of bias (WMD = −19.20; 95%CI −24.90 to −13.50; *P* < 0.001), low risk of bias trials (WMD = −14.55; 95%CI −22.47 to −6.64; *P* < 0.001) (Table 3). Between study heterogeneity was found for LDL (I^2^ = 96.1%). The heterogeneity disappeared when subgroup analysis was conducted on baseline LDL (< 100) (I^2^ = 56.6%, *P* = 0.075), health status including dyslipidemia (I^2^ = 54.9%, *P* = 0.065), metabolic syndrome (I^2^ = 0.00%, *P* = 0.348), and non-alcoholic fatty liver disease (I^2^ = 0.00%, *P* = 0.883) and low risk of bias trials (I^2^ = 40.4%, *P* = 0.187) (Table 3).

### Effect of BBR supplementation on HDL

A total of 34 effect sizes from 34 studies were included in the meta-analysis of the effect of BBR supplementation on HDL (Figure 2D) (36, 37, 40–42, 44, 47, 48, 50, 52–56, 58–63, 65–67, 71, 72, 74–76, 78, 79, 81, 84). BBR supplementation significantly increased HDL compared to placebo (WMD = 1.37 mg/dl; 95%CI, 0.41–2.23; *P* = 0.005). The subgroup analysis showed that the effect of BBR supplementation on HDL was significant in studies conducted on the baseline HDL < 40 mg/dl (WMD = 1.17 mg/dl; 95%CI, 0.08 to 2.27; *P* = 0.035), trial duration ≤ 8 weeks (WMD = 2.17; 95%CI, 0.10 to 4.23; *P* = 0.039), intervention dose >1 g/d (WMD = 1.81; 95%CI, 0.88 to 2.75; *P* < 0.001), obese (>30 kg/m^2^) (WMD = 4.85; 95%CI, 1.52 to 8.17; *P* = 0.004), type 2 diabetes (WMD = 1.65; 95%CI, 0.19 to 3.10; *P* = 0.026), and metabolic syndrome (WMD = 6.90; 95%CI, 2.42 to 11.37; *P* = 0.002), and category of risk of bias of trails, high risk of bias (WMD = 1.22; 95%CI 0.08 to 2.36; *P* = 0.035), low risk of bias trials (WMD = 5.46; 95%CI 0.93 to 9.99; *P* = 0.018) (Table 3). Between study heterogeneity was observed for HDL (I^2^ = 92.7%). The heterogeneity diminished when subgroup analysis was performed on health status including dyslipidemia (I^2^ = 47.9%, *P* = 0.104), metabolic syndrome (I^2^ = 60.8%, *P* = 0.078), and non-alcoholic fatty liver disease (I^2^ = 0.00%, *P* = 0.988) (Table 3).

### Effect of BBR supplementation on FBG

A total of 35 effect sizes from 35 studies were included in the meta-analysis of the effect of BBR supplementation on FBG (Figure 2E) (36–44, 47, 50, 54–56, 58–63, 65–67, 69, 70, 72, 74–78, 82–84). BBR supplementation significantly decreased FBG compared to placebo (WMD = −7.74 mg/dl; 95%CI, −10.79 to −4.70; *P* < 0.001). The subgroup analysis showed that the effect of BBR supplementation on FBG was significant in studies conducted baseline FBG ≥ 100 mg/dl (WMD = −10.61 mg/dl; 95%CI, −15.94 to −5.27; *P* < 0.001), trial duration >8 weeks (WMD = −10.83; 95%CI, −14.73 to −6.92; *P* < 0.001), intervention dose ≤ 1 g/d (WMD = −4.73 g/d; 95%CI, −8.75 to −0.71; *P* = 0.021) and >1 g/d (WMD = −9.98 g/d; 95%CI, −14.88 to −4.88; *P* < 0.001), normal (18.5–24.9 kg/m^2^) (WMD = −3.44; 95%CI, −5.75 to −1.13; *P* = 0.003), overweight (25–29.9 kg/m^2^) (WMD = −9.21; 95%CI, −12.90 to −5.52; *P* < 0.001), type 2 diabetes (WMD = −16.84; 95%CI, −24.51 to −9.17; *P* < 0.001), and non-alcoholic fatty liver diseases (WMD = −2.21; 95%CI, −4.41 to −0.02; *P* = 0.048), and category of risk of bias of trails, high risk of bias (WMD = −6.67; 95%CI −10.61 to −2.90; *P* = 0.001), moderate risk of bias (WMD = −13.56; 95%CI −26.81 to −0.31; *P* = 0.045) (Table 3). Between study heterogeneity was found for FBG (I^2^ = 97.0%). The heterogeneity diminished when the subgroup analysis was performed on BMI categories including normal BMI (I^2^ = 47.6%, *P* = 0.089), and obesity (I^2^ = 14%, *P* = 0.322) (Table 3).

### Effect of BBR supplementation on insulin

A total of 16 effect sizes from 16 studies were included in the meta-analysis of the effect of BBR supplementation on insulin (Figure 2F) (36, 38, 41, 44, 47, 56, 58, 60, 63, 67, 69, 72, 75, 76, 78, 83). BBR supplementation significantly decreased insulin compared to placebo (WMD = −3.27 mg/dl; 95%CI, −4.46 to −2.07; *P* < 0.001). The subgroup analysis showed that the effect of BBR supplementation on insulin was significant in studies conducted with trial duration ≤ 8 weeks (WMD = −3.74; 95%CI, −6.45 to −1.04; *P* = 0.007) and >8 weeks (WMD = −3.28; 95%CI, −5.01 to −1.54; *P* < 0.001), intervention dose ≤ 1 g/d (WMD = −2.54 g/d; 95%CI, −5.01 to −0.06; *P* = 0.044) and >1 g/d (WMD = −3.91; 95%CI, −5.58 to −2.24; *P* < 0.001), overweight (25–29.9 kg/m^2^) (WMD = −4.11; 95%CI, −5.87 to −2.35; *P* < 0.001), obese (>30 kg/m^2^) (WMD = −2.98; 95%CI, −4.66 to −1.29; *P* = 0.001), type 2 diabetes (WMD = −3.35; 95%CI, −4.98 to −1.72; *P* < 0.001), and others (WMD = −2.08; 95%CI, −3.74 to −0.42; *P* = 0.014), and category of risk of bias of trails, high risk of bias (WMD = −4.34; 95%CI −6.50 to −2.17; *P* < 0.001), moderate risk of bias (WMD = −1.90; 95%CI −2.42 to −1.38; *P* < 0.001). Between study heterogeneity was found for insulin (I^2^ = 95.3%). The heterogeneity diminished when the subgroup analysis was performed on the risk of bias, moderate risk of bias (I^2^ = 0.0%, *P* = 0.928) (Table 3).

### Effect of BBR supplementation on HbA1c

A total of 21 effect sizes from 21 studies were included in the meta-analysis of the effect of BBR supplementation on HbA1c (Figure 2G) (40, 41, 43, 47–49, 54, 56–58, 65, 67, 69, 72, 74–78). BBR supplementation significantly decreased HbA1c compared to placebo (WMD = −0.45%; 95%CI, −0.68 to −0.23; *P* < 0.001). The subgroup analysis showed that the effect of BBR supplementation on HbA1c was significant in studies conducted trial duration > 8 weeks (WMD = −0.61; 95%CI, −0.85 to −0.232; *P* < 0.001), intervention dose >1 g/d (WMD = −0.64; 95%CI, −0.92 to −0.37; *P* < 0.001), normal (18.5–24.9 kg/m^2^) (WMD = 0.53; 95%CI, 0.28 to 0.79; *P* < 0.001), overweight (25–29.9 kg/m^2^) (WMD = −0.41; 95%CI, −0.53 to −0.29; *P* < 0.001), obese (>30 kg/m^2^) (WMD = −0.94; 95%CI, −1.36 to −0.53; *P* < 0.001), type 2 diabetes (WMD = −0.51; 95%CI, −0.87 to −0.16; *P* = 0.004), non-alcoholic fatty liver disease (WMD = −0.34; 95%CI, −0.46 to −0.22; *P* < 0.001), and category of risk of bias of trails, high risk of bias (WMD = −0.52; 95%CI −0.77 to −0.27; *P* < 0.001) (Table 3). Between study heterogeneity was found for HbA1c (I^2^ = 92.5%). The heterogeneity disappeared when subgroup analysis was performed on BMI categories including normal BMI (I^2^ = 0.00%, *P* = 0.909), overweight (I^2^ = 41.6%, *P* = 0.057), health status (non-alcoholic fatty liver disease) (I^2^ = 41.7%, *P* = 0.180), moderate (I^2^ = 66.9%, *P* = 0.082) (Table 3).

### Effect of BBR supplementation on HOMA-IR

A total of 14 effect sizes from 14 studies were included in the meta-analysis of the effect of BBR supplementation on HOMA-IR (Figure 2H) (36, 38, 41, 44, 47, 48, 56, 58, 63, 67, 69, 72, 75, 78). BBR supplementation significantly decreased HOMA-IR compared to placebo (WMD = −1.04; 95%CI, −1.55 to −0.52; *P* < 0.001). The subgroup analysis showed that the effect of BBR supplementation on HOMA-IR was significant in studies conducted trial duration > 8 weeks (WMD = −1.13; 95%CI, −1.40 to −0.86; *P* < 0.001), intervention dose ≤ 1 g/d (WMD = −1.37; 95%CI, −2.12 to −0.62; *P* < 0.001) and >1 g/d (WMD = −0.77; 95%CI, −1.36 to −0.18; *P* = 0.010), normal (18.5–24.9 kg/m^2^) (WMD = −0.93; 95%CI, −1.73 to −0.14; *P* = 0.021), overweight (25–29.9 kg/m^2^) (WMD = −1.03; 95%CI, −1.50 to −0.56; *P* < 0.001), obese (>30 kg/m^2^) (WMD = −1.31; 95%CI, −1.90 to −0.73; *P* < 0.001), type 2 diabetes (WMD = −1.25; 95%CI, −1.62 to −0.88; *P* < 0.001) others (WMD = −0.62; 95%CI, −1.24 to −0.00; *P* = 0.047), and category of risk of bias of trails, high risk of bias (WMD = −1.12; 95%CI −1.59 to −0.65; *P* < 0.001), moderate (WMD = −1.10; 95%CI −1.18 to −1.02; *P* < 0.001) (Table 3). Between study heterogeneity was found for HOMA-IR (I^2^ = 99.1%). The heterogeneity diminished when subgroup analysis was performed on BMI categories (normal) (I^2^ = 71.9%, *P* = 0.059), and health status (non-alcoholic fatty liver disease) (I^2^ = 0.0%, *P* = 0.518), low risk of bias (I^2^ = 0.0%, *P* = 0.498) (Table 3).

### Effect of BBR supplementation on SBP

A total of 20 effect sizes from 20 studies were included in the meta-analysis of the effect of BBR supplementation on SBP (Figure 2I) (37, 40, 42, 43, 47, 48, 50, 54, 55, 58, 62, 63, 65, 69, 70, 78, 79, 82, 83, 85). BBR supplementation significantly decreased SBP compared to placebo (WMD = −5.46 mmHg; 95%CI, −8.17 to −2.76; *P* < 0.001). The subgroup analysis showed that the effect of BBR supplementation on SBP was significant in studies conducted baseline SBP < 120 mmHg (WMD = −2.93 mmHg; 95%CI, −4.09 to −1.76; *P* < 0.001), and ≥120 mmHg (WMD = −10.29; 95%CI, −16.75 to −3.82; *P* = 0.002), trial duration ≤ 8 weeks (WMD = −6.83; 95%CI, −11.98 to −1.68; *P* = 0.009) and > 8 weeks (WMD = −4.68; 95%CI, −7.99 to −1.36; *P* = 0.006), intervention dose ≤ 1 g/d (WMD = −3.85; 95%CI, −7.50 to −0.19; *P* = 0.039) and >1 g/d (WMD = −7.58; 95%CI, −11.79 to −3.36; *P* < 0.001), overweight (25–29.9 kg/m^2^) (WMD = −5.20; 95%CI, −8.48 to −1.92; *P* = 0.002), obese (>30 kg/m^2^) (WMD = −9.69; 95%CI, −15.77 to −3.60; *P* = 0.002), type 2 diabetes (WMD = −6.99; 95%CI, −11.29 to −2.68; *P* = 0.001), metabolic syndrome (WMD = −5.70; 95%CI, −8.49 to −2.91; *P* < 0.001), others (WMD = −3.76; 95%CI, −6.97 to −0.55; *P* = 0.022), and high risk of bias (WMD = −6.73; 95%CI −10.19 to −3.27; *P* < 0.001), moderate (WMD = −2.27; 95%CI −4.33 to −0.21; *P* = 0.030) (Table 3). Between study heterogeneity was found for SBP (I^2^ = 86.3%). The heterogeneity diminished when subgroup analysis was conducted on baseline SBP (I^2^ = 0.0%, *P* = 0.480), BMI categories (I^2^ = 0.0%, *P* = 0.363), and health status including dyslipidemia (I^2^ = 0.0%, *P* = 0.779), metabolic syndrome (I^2^ = 0.0%, *P* = 0.839), other health status (I^2^ = 21.1%, *P* = 0.281), and moderate risk of bias (I^2^ = 0.0%, *P* = 0.667) (Table 3).

### Effect of BBR supplementation on DBP

A total of 20 effect sizes from 20 studies were included in the meta-analysis of the effect of BBR supplementation on DBP (Figure 2J) (37, 40, 42, 43, 47, 48, 50, 54, 55, 58, 62, 63, 65, 69, 70, 78, 79, 82, 83, 85). The effect of BBR supplementation on DBP was non-significant (WMD = −2.74 mmHg; 95%CI, −5.63 to 0.15; *P* = 0.063). The subgroup analysis showed that the effect of BBR supplementation on DBP was significant in studies conducted on trial duration ≤ 8 (WMD = −3.12; 95%CI, −5.47 to −0.77; *P* = 0.009), and intervention dose >1 (WMD = −2.95; 95%CI, −4.90 to −1.00; *P* = 0.003), metabolic syndrome (WMD = −5.18; 95%CI, −6.91 to −3.45; *P* < 0.001) (Table 3). Between study heterogeneity was found for DBP (I^2^ = 94.9%). The heterogeneity diminished when subgroup analysis was conducted on BMI categories (I^2^ = 0.0%, *P* = 0.628), health status including dyslipidemia (I^2^ = 15.7%, *P* = 0.276), metabolic syndrome (I^2^ = 0.0%, *P* = 0.502), and moderate (I^2^ = 0.0%, *P* = 0.734) (Table 3).

### Effect of BBR supplementation on CRP

A total of nine effect sizes from nine studies were included in the meta-analysis of the effect of BBR supplementation on CRP (Figure 2K) (39, 42, 43, 46, 51, 59, 78, 81, 83). The effect of BBR supplementation on CRP was non-significant (WMD = 0.05; 95%CI, −0.59 to 0.68; *P* = 0.887). The subgroup analysis showed that the effect of BBR supplementation on CRP was significant in studies conducted on intervention dose ≤ 1 g/d (WMD = −0.56; 95%CI, −0.87 to −0.25; *P* < 0.001), BMI categories (WMD = −1.06; 95%CI, −1.77 to −0.34; *P* = 0.003) (Table 3). Between study heterogeneity was found for CRP (I^2^ = 97.4%) (Table 3).

### Effect of BBR supplementation on IL-6

A total of four effect sizes from four studies were included in the meta-analysis of the effect of BBR supplementation on IL-6 (Figure 2L) (39, 59, 78, 83). The effect of BBR supplementation on IL-6 was non-significant (WMD = −0.53; 95%CI, −1.11 to 0.05; *P* = 0.073). The subgroup analysis showed that the effect of BBR supplementation on IL-6 was significant in studies conducted on trial duration ≤ 8 weeks (WMD = −0.56; 95%CI, −1.21 to 0.08; *P* < 0.001), intervention dose ≤ 1 g/d (WMD = −0.55; 95%CI, −0.74 to −0.36; *P* < 0.001), and BMI categories (WMD = −0.56; 95%CI, −0.75 to −0.37; *P* ≤ 0.001) (Table 3). Between study heterogeneity was found for IL-6 (I^2^ = 94.7%). The heterogeneity diminished when subgroup analysis was conducted on intervention dose ≤ 1 g/d (I^2^ = 0.0%, *P* = 0.766) (Table 3).

### Effect of BBR supplementation on weight

A total of 21 effect sizes from 21 studies were included in the meta-analysis of the effect of BBR supplementation on weight (Figure 2M) (37, 40, 41, 45, 47, 49, 50, 54, 55, 58, 60, 61, 65, 67, 72, 78, 83, 84). BBR supplementation significantly decreased weight compared to placebo (WMD = −0.84; 95%CI, −1.34 to −0.34; *P* < 0.001). The subgroup analysis showed that the effect of BBR supplementation on weight was significant in studies conducted trial duration >8 weeks (WMD = −0.87; 95%CI, −1.44 to −0.31; *P* = 0.002), intervention dose >1 g/d (WMD = −1.52; 95%CI, −2.40 to −0.65; *P* = 0.001), overweight (WMD = −83; 95%CI, −1.19 to −0.47; *P* < 0.001), type 2 diabetes (WMD = −1.58; 95%CI, −2.52 to −0.64; *P* = 0.001) and non-alcoholic fatty liver disease (WMD = −1.63; 95%CI, −2.97 to −0.29; *P* = 0.017), high risk of bias (WMD = −1.02; 95%CI, −1.53 to −0.50; *P* < 0.001). Between study heterogeneity was found for the weight (I^2^ = 21.2%) (Table 3).

### Effect of BBR supplementation on BMI

A total of 24 effect sizes from 24 studies were included in the meta-analysis of the effect of BBR supplementation on BMI (Figure 2N) (36, 37, 40, 41, 44, 45, 47, 48, 54, 55, 58, 60–63, 67, 69, 72, 75, 78, 79, 82–84). BBR supplementation significantly decreased BMI compared to placebo (WMD = −0.25 kg/m^2^; 95%CI, −0.46 to −0.04; *P* = 0.020). The subgroup analysis showed that the effect of BBR supplementation on BMI was significant in studies conducted on overweight (25–29.9 kg/m^2^) (WMD = −0.27 kg/m^2^; 95%CI, −0.39 to −0.15; *P* < 0.001) (Table 3). Between study heterogeneity was found for BMI (I^2^ = 44.7%). The heterogeneity diminished when subgroup analysis was conducted on trial duration (I^2^ = 0.0%, *P* = 0.765), intervention dose (I^2^ = 12.6%, *P* = 0.316), BMI categories (I^2^ = 0.0%, *P* = 0.504), and health status including dyslipidemia (I^2^ = 0.0%, *P* = 0.577), metabolic syndrome (I^2^ = 0.0%, *P* = 0.446), and non-alcoholic fatty liver disease (I^2^ = 0.00%, *P* = 0.454), and moderate risk of bias (I^2^ = 0.00%, *P* = 0.449) (Table 3).

### Effect of BBR supplementation on WC

A total of 11 effect sizes from 11 studies were included in the meta-analysis of the effect of BBR supplementation on WC (36, 37, 40, 41, 48, 55, 60, 62, 72, 75, 84) (Figure 2O). BBR supplementation significantly decreased BMI compared to placebo (WMD = −1.77 kg/m^2^; 95%CI, −3.55 to 0.01; *P* = 0.005). The subgroup analysis showed that the effect of BBR supplementation on WC was significant in studies conducted on overweight (25–29.9 kg/m^2^) (WMD = −1.37 kg/m^2^; 95%CI, −2.71 to −0.03; *P* = 0.044), >1 g/d dose (WMD = −2.75 kg/m^2^; 95%CI, −3.72 to −1.77; *P* < 0.001) (Table 3). Between study heterogeneity was found for WC (I^2^ = 92.9%). The heterogeneity diminished when subgroup analysis was conducted on >1 g/d dose (I^2^ = 0.0%, *P* = 0.825), moderate risk of bias (I^2^ = 0.0%, *P* = 0.612) (Table 3).

### Effect of BBR supplementation on ALT

A total of 12 effect sizes from 12 studies were included in the meta-analysis of the effect of BBR supplementation on ALT (Figure 2P) (41, 49, 50, 52, 61, 63, 65, 73, 75, 80, 84). The effect of BBR supplementation on ALT was non-significant (WMD = −4.22; 95%CI, −8.75 to 0.31; *P* = 0.068) (Table 3). Between study heterogeneity was found for ALT (I^2^ = 92.3%) The heterogeneity diminished when subgroup analysis was conducted on trial duration ≤ 8 weeks (I^2^ = 0.0%, *P* = 0.433), intervention dose >1 g/d (I^2^ = 48.4%, *P* = 0.121), overweight (25–29.9 kg/m^2^) (I^2^ = 30.2%, *P* = 0.220), obese (>30 kg/m^2^) (I^2^ = 63.2%, *P* = 0.066), type 2 diabetes (I^2^ = 42.8%, *P* = 0.155), and other health status (I^2^ = 69.8%, *P* = 0.069) (Table 3).

### Effect of BBR supplementation on AST

A total of nine effect sizes from nine studies were included in the meta-analysis of the effect of BBR supplementation on AST (Figure 2Q) (41, 50, 52, 61, 63, 65, 75, 80, 84). The effect of BBR supplementation on AST was non-significant (WMD = −2.94; 95%CI, −8.68 to 2.81; *P* = 0.316). The subgroup analysis showed that the effect of BBR supplementation on AST was significant in studies conducted on other health statuses (WMD = 2.50; 95%CI, 0.97 to 4.03; *P* = 0.001) (Table 3). Between study heterogeneity was found for AST (I^2^ = 95.8%) The heterogeneity diminished when subgroup analysis was conducted on trial duration ≤ 8 weeks (I^2^ = 0.0%, *P* = 0.758), intervention dose >1 g/d (I^2^ = 0.0%, *P* = 0.910), BMI categories (I^2^ = 55.3%, *P* = 0.063), and type 2 diabetes (I^2^ = 0.0%, *P* = 0.992) (Table 3).

### Publication bias

**Figure 3:**
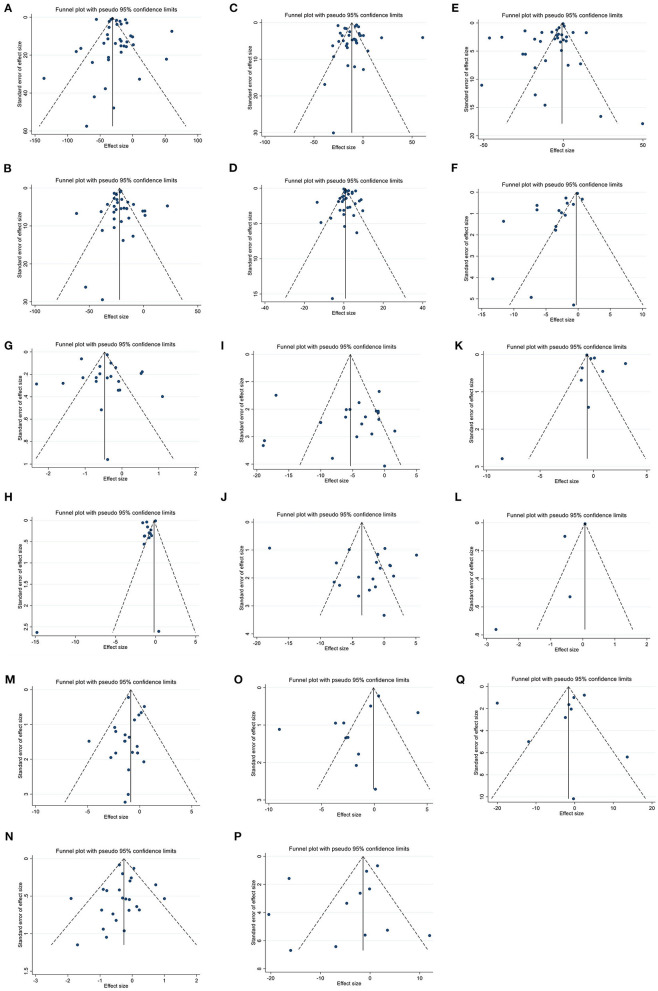
Funnel plots for the effect of berberine consumption on **(A)** TG (mg/dl); **(B)** TC (mg/dl); **(C)** LDL (mg/dl); **(D)** HDL (mg/dl); **(E)** FBG (mg/dl); **(F)** Insulin (mg/dl); **(G)** HbA1c (%); **(H)** HOMA-IR; **(I)** SBP (mmHg); **(J)** DBP (mmHg); **(K)** CRP (mg/L); **(L)** IL-6 (ng/L); **(M)** weight (kg); **(N)** BMI (kg/m^2^); **(O)** WC (cm); **(P)** ALT (U/L); and **(Q)** AST (U/L). TG, triglyceride; TC, total cholesterol; LDL, low-density lipoprotein; HDL, high-density lipoprotein; FBG, fasting blood glucose; HOMA-IR, homeostasis model assessment for insulin resistance; hemoglobin A1c, HbA1c; CRP, C-reactive protein; IL-6, interleukin 6; WC, waist circumference; ALT, alanine transaminase; AST, aspartate transaminase; SBP, systolic blood pressure; DBP, diastolic blood pressure; CI, confidence interval, weighted mean difference; WMD.

While the visual inspection of funnel plots showed slight asymmetries in funnel plots for all outcomes, no significant bias was detected based on Begg's for TG, TC, LDL, HDL, FBG, insulin, HbA1c, HOMA-IR, SBP, DBP, weight, BMI, WC, ALT and Egger's tests for CRP, IL-6, and AST (Figures 3A–Q).

### Meta-regression analysis

**Figure 4:**
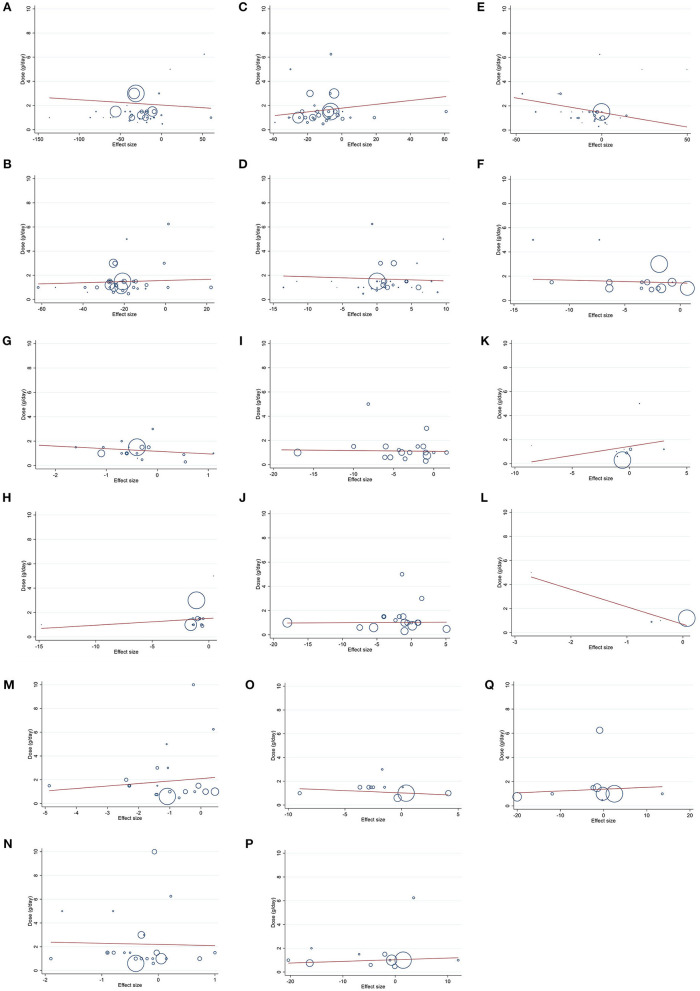
Linear dose-response relations between berberine consumption and absolute mean differences. Dose-response relations between dose (g/day) and absolute mean differences in **(A)** TG (mg/dl); **(B)** TC (mg/dl); **(C)** LDL (mg/dl); **(D)** HDL (mg/dl); **(E)** FBG (mg/dl); **(F)** Insulin (mg/dl); **(G)** HbA1c (%); **(H)** HOMA-IR; **(I)** SBP (mmHg); **(J)** DBP (mmHg); **(K)** CRP (mg/L); **(L)** IL-6 (ng/L); **(M)** weight (kg); **(N)** BMI (kg/m^2^); **(O)** WC (cm); **(P)** ALT (U/L); and **(Q)** AST (U/L). TG, triglyceride; TC, total cholesterol; LDL, low-density lipoprotein; HDL, high-density lipoprotein; FBG, fasting blood glucose; HOMA-IR, homeostasis model assessment for insulin resistance; hemoglobin A1c, HbA1c; CRP, C-reactive protein; IL-6, interleukin 6; WC, waist circumference; ALT, alanine transaminase; AST, aspartate transaminase; SBP, systolic blood pressure; DBP, diastolic blood pressure.

**Figure 5:**
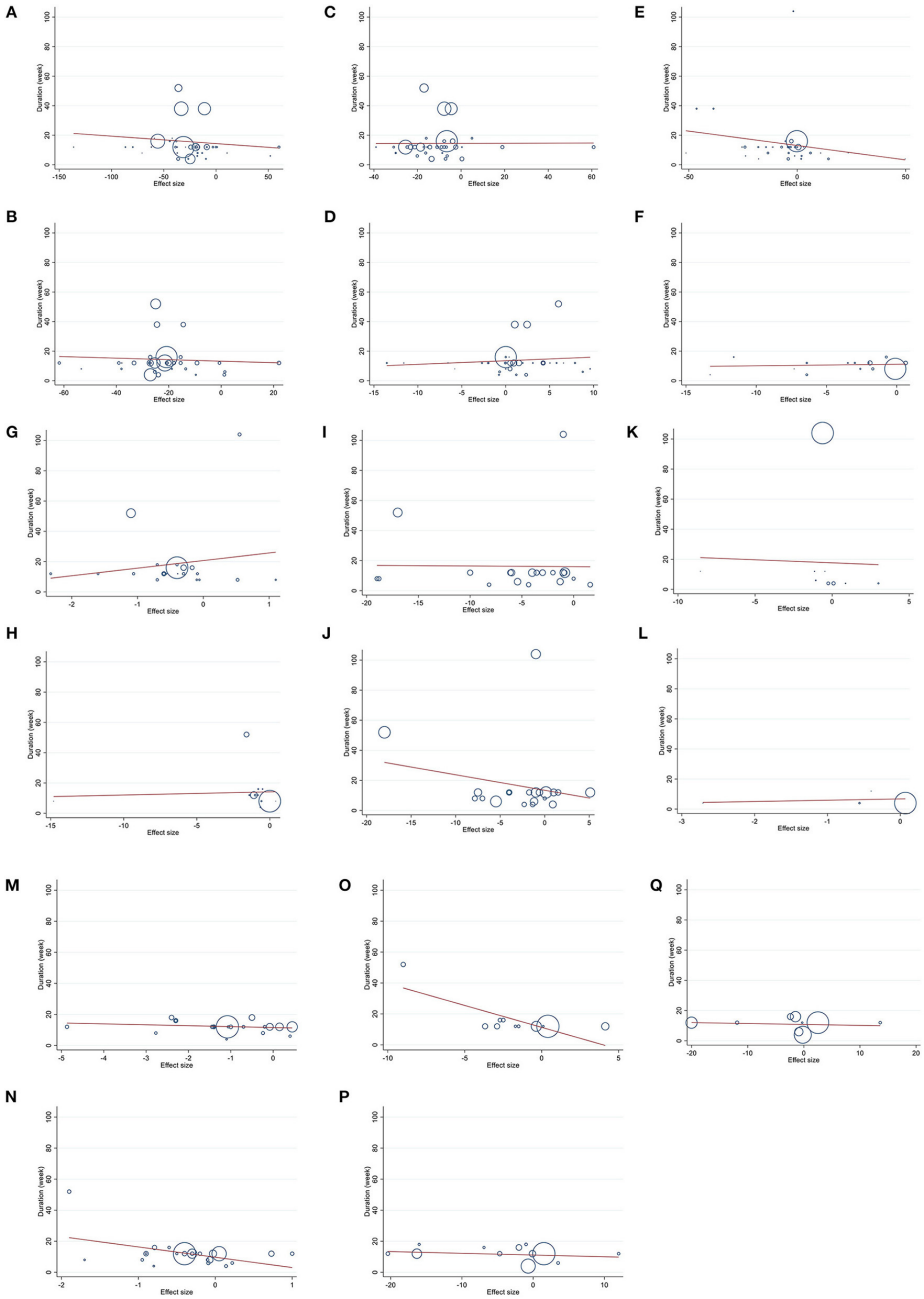
Linear dose-response relations between berberine consumption and absolute mean differences. Dose-response relations between duration of intervention (week) and absolute mean differences in **(A)** TG (mg/dl); **(B)** TC (mg/dl); **(C)** LDL (mg/dl); **(D)** HDL (mg/dl); **(E)** FBG (mg/dl); **(F)** Insulin (mg/dl); **(G)** HbA1c (%); **(H)** HOMA-IR; **(I)** SBP (mmHg); **(J)** DBP (mmHg); **(K)** CRP (mg/L); **(L)** IL-6 (ng/L); **(M)** weight (kg); **(N)** BMI (kg/m^2^); **(O)** WC (cm); **(P)** ALT (U/L); and **(Q)** AST (U/L). TG, triglyceride; TC, total cholesterol; LDL, low-density lipoprotein; HDL, high-density lipoprotein; FBG, fasting blood glucose; HOMA-IR, homeostasis model assessment for insulin resistance; hemoglobin A1c, HbA1c; CRP, C-reactive protein; IL-6, interleukin 6; WC, waist circumference; ALT, alanine transaminase; AST, Aspartate transaminase; SBP, systolic blood pressure; DBP, diastolic blood pressure.

Linear regression analyses were have done to examine if outcomes were affected by BBR doses (Figures 4A–Q) and intervention (Figures 5A–Q). A significant linear relationship between duration (weeks) and changes in BMI (coefficients = −6.64, *P*_linearity =_ 0.019) (Figure 5N) and WC (coefficients = −2.83, *P*_linearity_ = 0.006) (Figure 5O) was observed.

### Dose-response non-linear analysis

**Figure 6:**
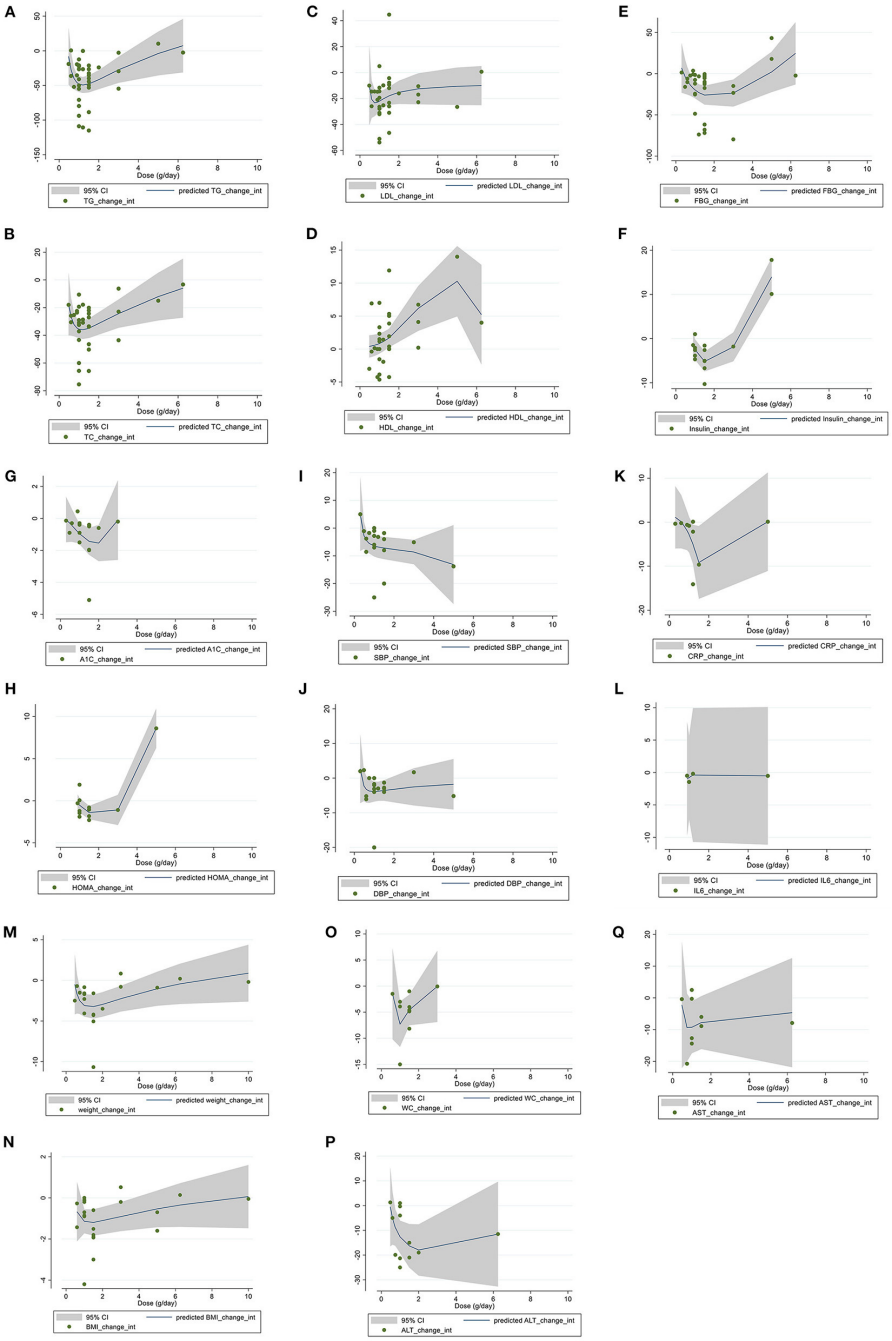
Non-linear dose-response relations between berberine consumption and absolute mean differences. Dose-response relations between dose (g/day) and absolute mean differences in **(A)** TG (mg/dl); **(B)** TC (mg/dl); **(C)** LDL (mg/dl); **(D)** HDL (mg/dl); **(E)** FBG (mg/dl); **(F)** Insulin (mg/dl); **(G)** HbA1c (%); **(H)** HOMA-IR; **(I)** SBP (mmHg); **(J)** DBP (mmHg); **(K)** CRP (mg/L); **(L)** IL-6 (ng/L); **(M)** weight (kg); **(N)** BMI (kg/m^2^); **(O)** WC (cm); **(P)** ALT (U/L); and **(Q)** AST (U/L). TG, triglyceride; TC, total cholesterol; LDL, low-density lipoprotein; HDL, high-density lipoprotein; FBG, fasting blood glucose; HOMA-IR, homeostasis model assessment for insulin resistance; hemoglobin A1c, HbA1c; CRP, C-reactive protein; IL-6, interleukin 6; WC, waist circumference; ALT, alanine transaminase; AST, Aspartate transaminase; SBP, systolic blood pressure; DBP, diastolic blood pressure.

**Figure 7:**
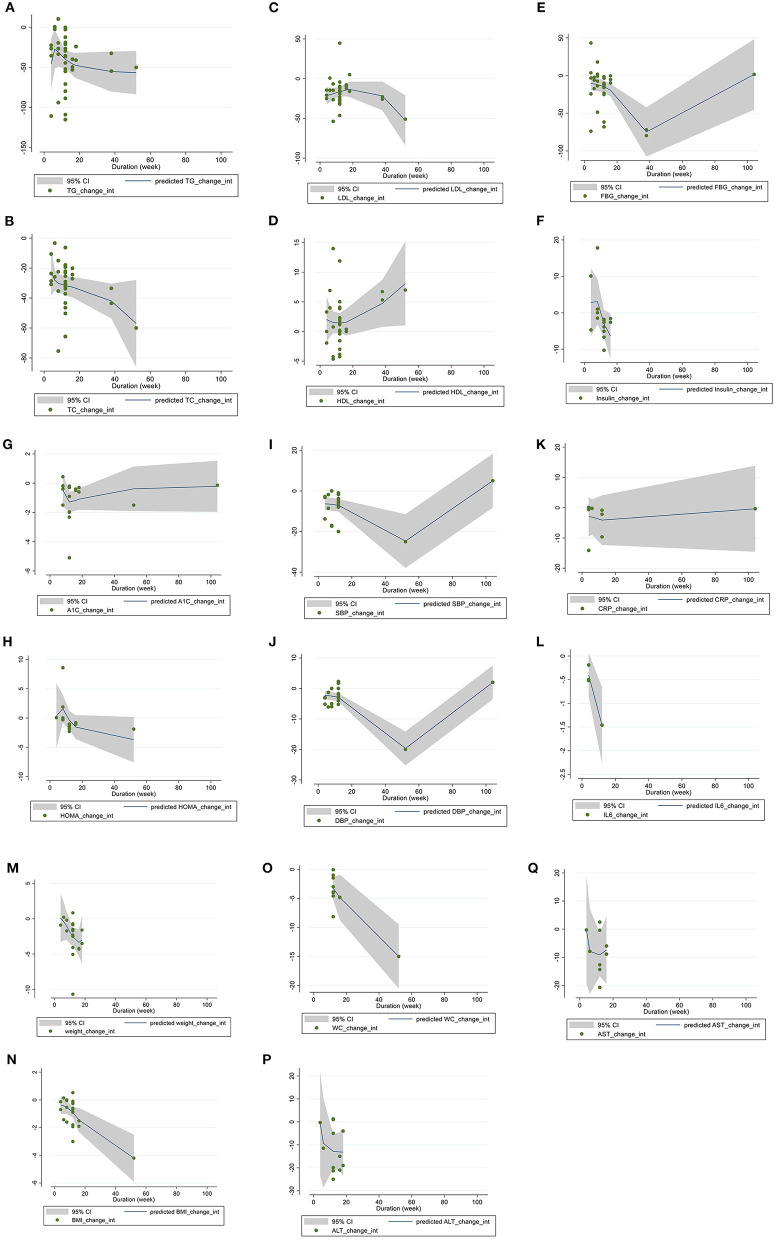
Non-linear dose-response relations between berberine consumption and absolute mean differences. Dose-response relations between duration of intervention (week) and absolute mean differences in **(A)** TG (mg/dl); **(B)** TC (mg/dl); **(C)** LDL (mg/dl); **(D)** HDL (mg/dl); **(E)** FBG (mg/dl); **(F)** Insulin (mg/dl); **(G)** HbA1c (%); **(H)** HOMA-IR; **(I)** SBP (mmHg); **(J)** DBP (mmHg); **(K)** CRP (mg/L); **(L)** IL-6 (ng/L); **(M)** weight (kg); **(N)** BMI (kg/m^2^); **(O)** WC (cm); **(P)** ALT (U/L); and **(Q)** AST (U/L). TG, triglyceride; TC, total cholesterol; LDL, low-density lipoprotein; HDL, high-density lipoprotein; FBG, fasting blood glucose; HOMA-IR, homeostasis model assessment for insulin resistance; hemoglobin A1c, HbA1c; CRP, C-reactive protein; IL-6, interleukin 6; WC, waist circumference; ALT, alanine transaminase; AST, aspartate transaminase; SBP, systolic blood pressure; DBP, diastolic blood pressure.

The non-linear dose response regression analysis have applied to assess whether outcomes were affected by BBR dose (Figures 6A–Q) and duration (Figures 7A–Q) of intervention. A significant non-linear effect of BBR dosage on serum concentrations of TG was found (coefficients = −238.29, *P*_non − linearity_ = 0.007). The effect was more prominent at a dose of 1 g/d (Figure 6A). A significant nonlinear effect of BBR dose was observed on serum concentration of TC (coefficients = 34.48, *P*_non − linearity_ = 0.013), while the association was more effective at a dose of 1 g/d (Figure 6B). A significant non-linear association was found between BBR dose (g/d) and HDL (coefficients = 0.50, *P*_non − linearity_ = 0.012), while the effect was more prominent at the dose of 5 g/d (Figure 6C). A significant non-linear effect of BBR dose (g/d) on levels of insulin was observed (coefficients = 1.09, *P*_non − linearity_ < 0.001), and the effective dose of BBR was more optimum at the dose of 1.8 g/d (Figure 6F). A significant non-linear association was found between BBR dose (g/d) and HOMA-IR (coefficients = 0.125, *P*_non − linearity_ < 0.001), while the association was more effective at a dose of 1.8 g/d (Figure 6H). A significant nonlinear association was found between BBR dose (g/d) and weight (coefficients = −15.20, *P*_non − linearity_ = 0.043), while the association was more prominent at the dose of 1.8g/d (Figure 6M).

Furthermore, a significant nonlinear association was found between the duration (weeks) of BBR use and serum concentration of FBG (coefficients = 1,179.70, *P*_non − linearity_ < 0.001), and the association was more effective at week 40 (Figure 7E). A significant non-linear effect of duration of BBR use (weeks) and DBP was observed (coefficients = 83.96, *P*_non − linearity_ < 0.001) while the effect was more prominent at 50 (Figure 7J). A significant nonlinear association was found between the duration (weeks) of BBR supplementation and WC (coefficients = 13.40, *P*_non − linearity_ = 0.005) and the association was more effective at week 50 (Figure 7O).

### Sensitivity analysis

By excluding each study, no study showed a significant impact on TG, TC, LDL, HDL, FBG, insulin, HbA1c, HOMA-IR, SBP, DBP, CRP, IL-6, AST, and weight. However, in the case of BMI, Chan et al. (40) showed a significant impact on overall effect size (WMD: −0.23, CI 95%: −0.47, *P* = 0.0003). Furthermore, regarding WC, León-Martínez et al. (55) (WMD: −1.88, CI 95%: −3.72, −0.03) and Mishra et al. (60) (WMD: −2.46, CI 95%: −4.20, −0.72) had a significant effect of overall effect size. Also, in terms of ALT, Zhao et al. (80) (WMD: −5.36, CI 95%: −10.01, −0.71) and Nejati et al. (61) (WMD: −4.80, CI 95%: −9.53, −0.06) showed a significant impact on overall effect size.

### GRADE assessment

The GRADE profile of BBR supplementation on the outcomes with the certainty in outcomes is shown in Table 4. The risk of bias for all the outcomes was a highly serious limitation, and a very serious limitation was found for inconsistency in the outcomes. The quality of evidence was reported low for TG, TC, LDL, HDL, FBG, insulin, HbA1c, HOMA-IR, and SBP and very low for CRP, IL-6, DBP, AST, ALT, and WC except for weight and BMI that was moderate.

**Table 4:** GRADE profile of berberine supplementation on cardiovascular risk factors in adults.

**Outcomes**	**Risk of bias**	**Inconsistency**	**Indirectness**	**Imprecision**	**Publication bias**	**WMD (95%CI)**	**Quality of evidence**
TG	High serious limitation	Very serious limitation ^a^	No serious limitation	No serious limitation	No serious limitation	−23.70 (−30.16, −17.25)	⊕⊕○○ Low
TC	High serious limitation	Very serious limitation ^a^	No serious limitation	No serious limitation	No serious limitation	−20.64 (−23.65, −17.63)	⊕⊕○○ Low
LDL	High serious limitation	Very serious limitation ^a^	No serious limitation	No serious limitation	No serious limitation	−9.63 (−13.87, −5.39)	⊕⊕○○ Low
HDL	High serious limitation	Very serious limitation ^a^	No serious limitation	No serious limitation	No serious limitation	1.37 (0.41, 2.33)	⊕⊕○○ Low
FBG	High serious limitation	Very serious limitation ^a^	No serious limitation	No serious limitation	No serious limitation	−7.74 (−10.79, −4.70)	⊕⊕○○ Low
Insulin	High serious limitation	Very serious limitation ^a^	No serious limitation	No serious limitation	No serious limitation	−3.27 (−4.46, −2.07)	⊕⊕○○ Low
HbA1c	High serious limitation	Very serious limitation ^a^	No serious limitation	No serious limitation	No serious limitation	−0.45 (−0.68, −0.23)	⊕⊕○○ Low
HOMA-IR	High serious limitation	Very serious limitation ^a^	No serious limitation	No serious limitation	No serious limitation	−1.04 (−1.55, −0.52)	⊕⊕○○ Low
SBP	High serious limitation	Very serious limitation ^a^	No serious limitation	No serious limitation	No serious limitation	−5.46 (−8.17, −2.76)	⊕⊕○○ Low
DBP	High serious limitation	Very serious limitation ^a^	No serious limitation	Serious limitation ^b^	No serious limitation	−2.74 (−5.63, 0.15)	⊕⊕○○ Very low
CRP	High serious limitation	Very serious limitation ^a^	No serious limitation	Serious limitation ^b^	No serious limitation	0.05 (−0.59, 0.68)	⊕⊕○○ Very low
IL-6	High serious limitation	Very serious limitation ^a^	No serious limitation	Serious limitation ^b^	No serious limitation	−0.53 (−1.11, 0.05)	⊕⊕○○ Very low
Weight	High serious limitation	No serious limitation	No serious limitation	No serious limitation	No serious limitation	−0.84 (−1.34, −0.34)	⊕○○○ Moderate
BMI	High serious limitation	No serious limitation	No serious limitation	No serious limitation	No serious limitation	−0.25 (−0.46, −0.04)	⊕○○○ Moderate
WC	High serious limitation	Very serious limitation ^a^	No serious limitation	Serious limitation ^b^	No serious limitation	−1.77 (−3.55, 0.01)	⊕○○○ Very low
ALT	High serious limitation	Very serious limitation ^a^	No serious limitation	Serious limitation ^b^	No serious limitation	−4.22 (−8.75, 0.31)	⊕⊕⊕○ Very low
AST	High serious limitation	Very serious limitation ^a^	No serious limitation	Serious limitation ^b^	No serious limitation	−2.94(−8.68, 2.81)	⊕⊕⊕○ Very low

a There is significant heterogeneity for TG (I^2^ = 96.6%), TC (I^2^ = 85.4%), LDL (I^2^ = 96.1%), HDL (I^2^ = 92.7%), FBG (I^2^ = 97.0%), Insulin (I^2^ = 95.3%), HbA1C (I^2^ = 92.5%), HOMA-IR (I^2^ = 99.1%), SBP (I^2^ = 86.3%), DBP (I^2^ = 94.9%), CRP (I^2^ = 97.4%), IL-6 (I^2^ = 94.7%), WC (I^2^ = 92.9%), ALT (I^2^ = 92.3%), and AST (I^2^ = 95.8%).

b There is no evidence of significant effects of berberine consumption on DBP, CRP, IL-6, WC, ALT, and AST.

⊕ shows +1 quality evidence that for every serious limitation, one of these quality evidences is lost.

## Discussion

This paper presents a comprehensive systematic review and dose-response meta-analysis of the effects of BBR supplementation on cardiovascular risk factors. The results showed that BBR supplementation can significantly lower TC, TG, LDL, FBG, insulin, HbA1c, HOMA-IR, SBP, weight, BMI, and WC, and can elevate HDL. According to the subgroup analysis, BBR supplementation in participants with normal BMIs (18.5–24.9) was ineffective for changing TG, TC, LDL, HDL, insulin, SBP, weight, BMI, and WC. The significant effects of BBR on HDL and WC were only seen in doses of more than 1 g/day, on FBG and HOMA-IR in the durations of more than 8 weeks, and on HbA1c and weight in both mentioned higher subgroups of dose (>1 g/d) and duration (>8 weeks). Moreover, BBR was significantly effective in alleviating cardiovascular risk factors, mainly in subgroups with impaired metabolic health such as NAFLD, type 2 diabetes, and metabolic syndrome. In addition, BBR was effective for the improvement of LDL, HDL, and FBG only in subgroups with abnormal ranges (HDL ≤ 40, LDL > 100 mg/dl, and FBG > 100 mg/dl). In the non-linear dose-response analysis, the optimum dose for BBR was 1 g/day for TG, TC, and weight, 1.8 g/day for insulin and HOMA-IR, and 5 g/day for HDL. The most effective duration was 40 weeks for FBG and 50 weeks from beginning of BBR supplementation for DBP and WC.

### Effects of BBR on FBG

BBR, a plant isoquinoline alkaloid with a long history of medical use (87), reduced FBG, insulin levels, HOMA-IR, and HbA1c in this meta-analysis significantly, and has been suggested to be significantly beneficial for the improvement of blood glucose and insulin resistance by other different meta-analyses over time (15, 16, 88, 89). Discussing the most recent studies, Ye et al., have shown in a meta-analysis of 18 clinical trials in 2021 that BBR consumption affects FBG, and HOMA-IR improvement (16). Another meta-analysis of 46 RCTs by Guo et al. (15), confirmed these results on FBG and HOMA-IR and added that 2-h postprandial blood glucose tests, fasting blood insulin, and HbA1c can be improved as well. These two studies have a good quality since they have done the risk of bias assessment, subgroup analysis, and sensitivity analysis. However, neither of them implemented dose-response analysis, which is done in this study. BBR has been known as comparable to metformin (90) and suggested as becoming an alternative to metformin in people with poor socioeconomic status (88). These effects can be owing to the activation of adenosine monophosphate-activated protein kinase (AMPK) following BBR consumption, which leads to the improvement of insulin sensitivity (16), promotion of the glucose transporters' levels (GLUT-4 and GLP-1) (16, 91), and an increase in insulin receptor expression through protein kinase C-dependent upregulation of its promoter (77, 92).

According to the subgroup analysis, it seems that BBR needs a supplementation duration of more than 8 weeks to reduce FBG, HbA1c, and HOMA-IR, and a dose of more than 1 gram per day to reduce HbA1c. This can be owing to the low bioavailability (< 1%) of this substance (93). Higher doses and duration may enhance the intestinal uptake leading to more effective outcomes. Moreover, the fact that changes in HOMA-IR (94) and HbA1c (95) test results are time-consuming and occur gradually over time may justify the above results. BBR was effective in reducing FBG only in the subgroup of FBG ≥100 which can be because of the induction of higher insulin secretion in hyperglycemia by BBR, as explained by a previous study (86). Another reason can be the anti-inflammatory properties of BBR (18, 93, 96) that result in FBG reduction only when it exceeds its normal range. Hyperglycemia induces oxidative stress (97) and BBR can act against the consequential inflammation. In addition, subgroup analysis showed that the significant results can be seen only in the subgroups with unhealthy metabolic status. This evidence can also be justified by the anti-inflammatory properties of BBR (18, 93, 96). Moreover, risk of bias subgroup analysis has shown even in high risk of bias trials decreasing effect remained for all glycemic markers and even in most of them in moderate risk apart from HbA1c. Of course, the absence of this significance can probably be attributed to the small number of studies in this subgroup (*n* = 2).

### Effects of BBR on lipid profile

This meta-analysis showed a significant effect of BBR on TG, TC, LDL, and HDL. In line with this study, all the previous meta-analyses in different years have shown beneficial effects of BBR on lipid profile improvement (12, 15, 16, 98–100). Two recent meta-analyses in 2021, done by Ye et al. (16) on 18 RCTs and by Guo et al. (15) on 46 RCTs have employed a high-quality methodology. However, this study, like other previous studies, did not do any dose-response analysis, which is presented in this study. BBR can influence the lipid profile by some main mechanisms. As mentioned before, BBR can activate AMP-activated protein kinase (AMPK). This activation leads to a reduction in fat production and changes fat accumulation to fat decomposition (16). The influence on lipid profile may also be due to intestinal absorption limitations and an increase in fecal cholesterol excretion following BBR consumption (90, 101).

In subgroup analysis, the reduction in TG and TC was significant only in participants with overweight (BMI: 25–29.9) and obesity (BMI ≥ 30), the significant reduction in LDL was only in the overweight subgroup, and the significant increase in HDL was only in the obese group. Moreover, the subgroups with unhealthy metabolic status and with abnormal LDL (≥100 mg/dl) and HDL (< 40 mg/dl) responded significantly to this supplementation. All these conditions are linked to the secretion of inflammatory mediators and may benefit from anti-inflammatory substances (102–104). As an anti-inflammatory agent, BBR is thought to inhibit the PI3K/AKT signaling pathway (93), suppress nuclear factor kappa B (NF-kB) signaling pathway (93, 96), and lower CRP, IL-6, and tumor necrosis factor-alpha (TNF-α) levels (18). Risk of bias subgroup analysis has shown that even in high and moderate risk of bias trials, the decreasing effect remained for all lipid profiles apart from HDL in moderate risk of bias. Of course, the absence of this significance can probably be attributed to the small number of studies in this subgroup (*n* = 5).

### Effects of BBR on anthropometric measures

The present study demonstrated a significant effect of BBR on weight, BMI, and WC. Two meta-analyses by Asbaghi et al. and Xiong et al., similar to this study, revealed the significant influence of BBR supplementation on the reduction of BMI and WC (13, 14). The anti-obesity effects of BBR can be owing to some reasons. First, BBR induces thermogenic effects through the AMPK-PRDM16 axis and brown adipocyte differentiation, leading to more energy expenditure (105). Second, it can modulate the gene expression of some factors involved in adipogenesis like peroxisome proliferator-activated receptor γ (PPARγ), cAMP-response element-binding protein (CREB), GATA-2, and GATA-3 (13, 91). It is also suggested that BBR can decrease the size and number of droplets of lipids in some specific regions of the body (90). Nevertheless, unlike the study by Asbaghi et al. and Xiong et al. could not see a significant change in weight after BBR intake. The third meta-analysis of 12 trials by Amini et al. in the same year could not see any significant reduction in BMI, WC, and weight following BBR supplementation (91). However, they reported a significant reduction in the waist-to-hip ratio (WHR) (91). These controversies highlight the need for a new conclusive meta-analysis.

According to the subgroup analysis, the only BMI category in which the reduction in weight, WC, and BMI was significant was the overweight (BMI: 25–29.9). The number of trials included in this category was more than 3-folds that of the obese (BMI 30) and normal weight (BMI: 18.5–24.9) categories combined. We may see significant results in other BMI subgroups if the sample size was more. Doses of more than 1 g/day were effective for WC and BMI reduction and a duration of more than 8 weeks was effective for weight loss, which can be attributed the aforementioned low bioavailability of BBR (93). In a risk of bias subgroup analysis, it was shown in high risk of bias trials, the decreasing effect remained for weight but not for BMI. However, it seems that although BBR may affect weight, it does not have a statistically significant and considerable effect (WMD = −0.28) on BMI. However, there is a possibility of a lack of sample size and power in this subgroup.

### Effects of BBR on blood pressure

The present study reports a significant reduction in SBP but a non-significant change in DBP following BBR supplementation. Regarding the previous studies on the effect of BBR on BP, a systematic review done in 2021 by Suadoni et al. reported that the evidence was not enough, of good quality, and suitable duration to report any significant effects (19), and a meta-analysis in 2015 by Lan et al. reported a non-significant result for this relationship on patients with type 2 diabetes (88). Another meta-analysis of 12 RCTs in 2021 showed that a supplement called Armolipid Plus, whose ingredients are BBR plus 5 other substances, was not effective in imposing changes in SBP and DBP (106). To reach a conclusive result, this meta-analysis comprehensively evaluates BBR's effects on different cardiovascular risk factors in different sub-groups, with dose-response analyses and with more included clinical trials.

Regarding the subgroup analysis, SBP was significantly reduced in all subgroups despite normal BMI, dyslipidemia, and NAFLD, in which the included trials were only 1 or 2 studies. DBP did not change in the majority of subgroups, despite being significantly lower in the intervention dose of >1. This finding highlights the need for more well-designed RCTs in the future with higher intervention doses. In risk of bias subgroup analysis has shown that in high and moderate risk of bias trials, the decreasing effect is constant for SBP.

### Effects of BBR on inflammatory markers

This meta-analysis could not see any significant changes in two main inflammatory markers, CRP and IL-6, following the supplementation with BBR that was not expected regarding the anti-inflammatory properties of BBR. This result is in contrast with the meta-analysis of 12 RCTs by Asbaghi et al. (13) that found a significant effect of this agent on CRP levels. Another previous meta-analysis of five non-heterogeneous RCTs by Beba et al. (17) supported the hypothesis of CRP reduction after BBR supplementation. Guo et al. have done another meta-analysis of 46 studies in 2021 and have found an effective reduction in IL-6, TNF-α, and CRP following BBR intake (15). A meta-analysis of 52 RCTs by Lu et al. (18) reported a significant BBR-induced reduction in these two inflammatory markers (CRP and IL-6). However, the participants of this study were only Chinese people, and the result should not be generalized (18). Generally, different factors can justify the contradictory results between the studies such as different races, genetics, sex, or age range of participants, different study durations, sampling methods; supplement form or dose, and other reasons that cause heterogeneity. Moreover, in the present analysis, only 4 studies were included for IL-6 and 9 studies were included for CRP hence the sample size was small.

### Effects of BBR on liver enzymes

Similar to two previous meta-analyses, one from 12 RCTs by Asbaghi et al. (13), and another from 5 trials by Mohtashaminia et al. (107), our results did not show any significant effect of BBR on liver function enzymes (ALT and AST). The effect of BBR on liver function enzymes was not seen in either of the subgroups.

In the non-linear dose-response analyses, we found that the approximate optimum dose for BBR supplementation for the reduction of TG, TC, and weight is 1 g/day. This dose is 1.8 g/day for insulin and HOMA-IR, and 5 g/day for HDL improvement. The most effective duration for BBR intake was 40 weeks for FBG and 50 weeks for DBP and WC from beginning of supplementation. Although the plasma concentration of BBR tends to be low owing to its poor oral absorption and bioavailability, its concentration in different tissues usually remains high (108). The pharmacokinetic profile of BBR indicated that its concentration in most tissues was higher than in plasma 4 h after administration (109). This characteristic of BBR may cause saturation of the body with it at high doses and durations and can be the reason why the supplementation of more than a specific dose or duration seems to be pointless in this analysis.

BBR has attracted many scientists' attention owing to its ameliorative effects on CVD risk factors (6, 7, 16, 77). The mechanisms by which BBR affects metabolic health are diverse and well-defined. BBR is suggested to upregulate the expression of LDL receptors in the human hepatoma cell line (HepG2) and to inhibit both cholesterol and TG synthesis in the liver, dose-dependently (110). This effect of BBR on lipid synthesis is mediated by the mitogen-activated protein kinase (MAPK/ERK) pathway (110), and can also be owing to the decrease in proprotein convertase subtilisin/kexin type 9 (PCSK9) mRNA. PCSK9 downregulates the LDL receptor (LDLR) and BBR acts against it (111). Another mechanism of action for BBR could be that it is an agonist for AMPK, a fuel gauge. This activation leads to the inhibition of cholesterol and TG synthesis by inactivating two enzymes, β-Hydroxy β-methylglutaryl-CoA (HMG-CoA) and ACC (acetyl-coenzyme A carboxylase) (110). AMPK activation also increases energy production hence normalizing the imbalance between glucose, lipid, and energy (16). This activation can also impose anti-inflammatory effects (112) and can speed up the transport of glucose in the serum by promoting glucose transporter type 4 **(**GLUT4) translocation (113). It is proposed by Zhang et al. that BBR can also increase the expression of the insulin receptor in a variety of human cells in type 2 diabetic patients (77). Li et al. induced hyperlipidemia in hamsters by feeding them with a high-fat diet and assessed the effect of BBR supplementation on this hyperlipidemia. The excretion of cholesterol to the liver, bile, and feces was promoted following BBR intake in hyperlipidemic hamsters but not in the normal group (114). Therefore, BBR seems to be a multi-targeted lipid-lowering agent. BBR, as an anti-inflammatory agent, is suggested to inhibit the phosphoinositide 3-kinase (PI3K)/AKT signaling pathway which reduces the secretion of pro-inflammatory cytokines or mediators in cardiomyocytes and serum, such as IL-6, Interleukin 1 beta (IL1β), CRP, and TNF-α (18, 93), and it can also suppress nuclear factor kappa B (NF-kB) signaling pathway (93, 96). Moreover, BBR induces thermogenic effects through the AMPK-PRDM16 axis that induces brown adipogenesis, leading to more energy expenditure (105). BBR can also suppress the expression of some factors involved in adipogenesis like PPARγ (115), CREB (116), GATA-2, and GATA-3 (117). It is also suggested that BBR can decrease the size and number of droplets of lipids in the 3T3-L1 adipocyte cell line (90). The other anti-diabetic mechanism of BRB is related to the modulating of gut microbiota (118). This agent works topically in the gastrointestinal tract as an antimicrobial agent to act against pathogens and inhibit their growth and block their adhesion to epithelial cells (118). The other protective role of BRB for the cardiovascular system can be the anti-hypertensive effects owing to its impact on vasodilation in middle cerebral arteries in rats (119), and its action against the renin-angiotensin system in rats (120). BBR attenuated ischemic-induced arrhythmias in diabetic rats *via* recovering depressed I (to) and I (Ca) currents (121).

This meta-analysis has various strengths and limitations. one of the most important strengths of this study can be considered the most comprehensive meta-analysis to date regarding the relationship between BBR and all different cardiovascular risk factors with a dose-response analysis with the larger sample size compared to the previous similar meta-analysis (13, 14, 16, 91, 99). Studies were included based on inclusion criteria, with varying individuals, which provides the possibility of subgroup analysis. The randomized and placebo-controlled design of all included trials and the double-blind design of most of them can also be other strengths. Another point to be mentioned is that the participants of the included trials were from different nations, of different ages and sexes, and with different existing morbidities such as NAFLD, metabolic syndrome, etc. This may enhance heterogeneity but can also make the results admissible to be generalized. No limitations on language and time for including studies. In addition, sensitivity tests in this study were used to identify potential sources of heterogeneity among trials. GRADE tools for quality assessment of studies and subgroup analysis especially for risk of bias have done. However, some limitations should be considered. Although all studies used randomization, information on allocation concealment, randomization efficiency, and withdrawal was not consistently disclosed. In the approach of statistical analysis, the control of covariate and confounding variables was not done in all studies. Small study sample sizes made randomization's capacity to lessen the potential effects of confounding variables difficult. The included studies were significantly heterogeneous. Regarding the considerable number of the included studies, the types of measurements for outcomes could be different. Intra assay coefficient of variation and inter-assay variability for biochemical kits in different studies might lead to different results. Same thing about, the anthropometric indices were measured by different scales and differently trained persons in the included studies. In addition, the blood pressure had been taken in different positions (seated or standing posture, supine position). Different sources of BBR supplementation in studies were used in the trials. In addition, in the analyses for liver enzymes and inflammatory markers, the number of included trials was small, hindering reliable results. Lastly according to risk of bias assessment, most of the included RCTs in this study has a high risk of bias. This highlights the need for more well-designed clinical trials in the future.

## Conclusion

This systematic review and dose-response meta-analysis found a significant improvement in lipid profiles, insulin resistance, and anthropometric measures associated with BBR supplementation. However, no significant changes have been observed in liver enzymes or inflammatory markers. Therefore, BBR may be an effective supplement for the improvement of metabolic syndrome and cardiovascular risk. To comprehend how BBR affects these outcomes in people, mechanistic research, homogeneous RCTs, and future investigations are required.

## Data availability statement

The original contributions presented in the study are included in the article/supplementary material, further inquiries can be directed to the corresponding authors.

## Author contributions

MZam designed the study. MZam and OA developed the search strategy and assessed the risk of bias in the meta-analyses. MZam, MN-S, and OA extracted the data and conducted the analyses. SH and MZar drafted the manuscript. FS, OA, and MN-S interpreted the results. FS and OA revised the manuscript. All authors read and approved the final manuscript.

## Conflict of interest

The authors declare that the research was conducted in the absence of any commercial or financial relationships that could be construed as a potential conflict of interest.

## Publisher's note

All claims expressed in this article are solely those of the authors and do not necessarily represent those of their affiliated organizations, or those of the publisher, the editors and the reviewers. Any product that may be evaluated in this article, or claim that may be made by its manufacturer, is not guaranteed or endorsed by the publisher.

## Abbreviations

CVD: Cardiovascular disease
WHO: World Health Organization
BBR: Berberine
CRP: C reactive protein
BMI: body mass index
PICO: Participant, Intervention, Comparison/Control, Outcome
TG: triglyceride
TC: total cholesterol
LDL: low-density lipoprotein
HDL: high-density lipoprotein
FBG: fasting blood glucose
HbA1c: hemoglobin A1c
HOMA-IR: homeostasis model assessment-insulin resistance
SBP: systolic blood pressure
DBP: diastolic blood pressure
CRP: C-reactive protein
IL-6: interleukin-6
WC: waist circumference
BMI: body mass index
AST: Aspartate transaminase
ALT: Alanine transaminase
GRADE: (Grading of Recommendations Assessment, Development, and Evaluation), WMD, weighted mean difference
AMPK: AMP-activated protein kinase
PI3K: phosphoinositide 3-kinase
NF-kB: nuclear factor kappa B
TNF-α: tumor necrosis factor alpha
PPARγ: peroxisome proliferator-activated receptor γ
CREB: cAMP-response element-binding protein
WHR: waist to hip ratio
HepG2: human hepatoma cell line
MAPK: mitogen-activated protein kinase
PCSK9: proprotein convertase subtilisin/kexin type 9
LDLR: LDL receptor
HMG-CoA: β-hydroxy β-methylglutaryl-CoA
ACC: acetyl-coenzyme A carboxylase
GLUT4: glucose transporter type 4
IL1β: Interleukin 1beta
NF-kB: nuclear factor kappa B
CREB: cAMP-response element-binding protein.
